# The Impact of Parental Detention on the Psychological Wellbeing of Palestinian Children

**DOI:** 10.1371/journal.pone.0133347

**Published:** 2015-07-17

**Authors:** Amer Shehadeh, Gerrit Loots, Johan Vanderfaeillie, Ilse Derluyn

**Affiliations:** 1 Research group Interpersonal, Discursive and Narrative Studies (IDNS) & Centre for Children in Vulnerable Situations (CCVS), Vrije Universiteit Brussel, Brussels, Belgium; 2 Ministry of Education, Directorate of Education, Bethlehem, Palestine; 3 Universidad Católica Boliviana “San Pablo” La Paz, Instituto de Investigaciones en Ciencias del Compartamiento (IICC), Departamento de Psicología, La Paz, Bolivia; 4 Department of Clinical and Lifespan Psychology, Vrije Universiteit Brussel, Brussels, Belgium; 5 Department of Social Welfare Studies & Centre for Children in Vulnerable Situations, Ghent University, Gent, Belgium; National Center of Neurology and Psychiatry, JAPAN

## Abstract

**Background:**

Since 1967, the Palestinian Occupied Territories are marked by a political conflict between Palestinians and Israel. During this conflict, about one fifth of the Palestinian population has been detained; about one quarter of these are parents. Although we know that father’s incarceration might impact their children’s psychological wellbeing, little is known about the impact of father’s imprisonment on young children (under 11 years old), and when the incarceration is framed in contexts of political conflict. Therefore, this study aimed at gaining insight into the impact of parental detention on young children’s psychological wellbeing, and the impact of witnessing the detention process itself.

**Methods:**

Based on the list of imprisoned Palestinian men with children living in the West Bank, a group of 79 (3- to 10-years old) children was randomly composed. Above, through schools and health centers, a comparison sample of 99 children who didn’t experience imprisonment of a family member was selected. Mothers of these children completed two cross-culturally validated questionnaires on their children’s psychological wellbeing, the UCLA-PTSD-Index and the Strength and Difficulties Questionnaire (SDQ).

**Results:**

Results showed higher levels of PTSD and general mental health problems associated with father’s capturing. Above, when the children watched the arrest process of their fathers, scores still increased further. Younger children tended to show higher SDQ scores, and children living in villages reported higher posttraumatic stress scores compared to children living in urban areas or refugee camps. Little gender differences were found.

**Conclusion:**

This study shows the important impact of parental detention on the psychological wellbeing for young children and urges for more psychological care and support for family members – in particular children – of detainees.

## Introduction

Detention of fathers largely impacts the psychological wellbeing of the children involved, with a large range of emotional and behavioral problems, such as posttraumatic stress disorder (PTSD), bedwetting, nightmares and anxiety, as possible consequences [1–9]. Other studies refer to, amongst other problems, attention deficits [10], antisocial behavior and violence [11], hopelessness [12], and feelings of rejection and guilt [13]. Above, within their social networks, children of arrested fathers are also confronted with feelings of shame and experiences of stigmatization [14], and therefore become more isolated from peers [13].

Some studies suggest that the impact of fathers’ detention onto children’s psychological and social wellbeing differs according to gender, with boys elaborating more externalizing problems and girls rather internalizing symptoms [10] although not all studies found gender differences[15,16]. Also, some studies report age differences, either in prevalence or in type of emotional problems [8,15], but also here, findings are not conclusive [17], and there is a paucity of literature investigating the impact of fathers’ detention onto the wellbeing of children who are younger than 11 years old.

Most of these studies have focused on children whose fathers were arrested because of particular criminal facts, and did not consider children whose fathers were arrested because of political reasons or within contexts of ongoing political conflicts. In these situations, the impact of arrest and detention can largely differ from that in contexts of individual crimes: Detainees and also their family members are seldom informed about the specific reason for detention, and the detention happens in particular circumstances (with, e.g., surrounding of houses, all family members forced to leaving the house, blindfolding of detainees,…). Further, detention in contexts of political conflict might be less associated with negative social views, also towards family members, compared to incarceration following individual criminal acts.

The POT is such a particular context, characterized by an ongoing conflict between Israeli and Palestinian people since the beginning of the nineteenth century. In 1967, the Palestinian territories—West Bank, East Jerusalem and the Gaza strip—were occupied by Israel. From that date on, a political conflict is going on between Israeli and Palestinians, a political conflict that has passed through ups and downs, with the most violent periods during the first (1987–1993) and the second Intifada (2000–2005) [18–19].

Imprisonment of Palestinian citizens by Israeli soldiers in the POT is throughout this conflict a daily phenomenon. In 2012, about nine persons were daily arrested [18], and, overall, since 1967, more than 20% (700,000 to 800,000 persons) of the Palestinian population experienced detention. These periods of detention lasted from 18 days (period of investigation) till lifelong. At the beginning of 2012, 4,500 Palestinians were in prison, including 280 administrative detentions without charge for at least six months and 532 prisoners sentenced to life [18]. About 89% (n = 4,006) of all prisoners are from West Bank, 494 (11%) from Gaza and East Jerusalem; 27.8% (n = 1,250) of these detainees are married and have children (table 1) [18,20]. So, an important number of Palestinian children are confronted with the imprisonment of their father.

**Table 1:** Demographic characteristics of Palestinian captives in 2012 [18].

Number of married prisoners (n = 1,250)	Sentence (years)
650	0–9
355	10–20
83	21–29
17	30–39
3	40–49
1	60–69
186	ever lasting

Although a large number of studies investigated the impact of the Israeli-Palestinian protracted conflict on Palestinian children’s psychological wellbeing [15,16,21–24], there are—as far as we know—no studies investigating the particular impact of paternal imprisonment onto Palestinian children. Furthermore, there seems to be no research on the impact of the circumstances of father’s arrest on children’s psychological wellbeing. Although it is well documented that witnessing a traumatic event can impact a person’s later mental health [3,6,14,25–28], the circumstances of a parent’s imprisonment on children have not been investigated. In the POT, Palestinian individuals are often arrested at checkpoints on the street, and frequently in their houses during night, while family members are sleeping. Usually, Israeli soldiers surround the house, knock with their guns on the doors, sometimes blow up doors or launch tear gas and sound bombs. The arrested family members are usually handcuffed with plastic cuffs and blindfolded [18,29].

The aim of this study is twofold: First, we investigate the impact of father’s imprisonment on the psychological wellbeing of young (<11 years old) Palestinian children, through comparing this group with a similar group of Palestinian children whose father has not been imprisoned. Next, we study the psychological consequences of witnessing the capturing of the fathers by comparing the children who saw the arrest of their fathers with those who did not watch it. Given the participants’ young ages, we will hereto rely onto the mother’s assessment of the child’s psychological wellbeing.

## Methods

### Ethics statement

This study was approved by the Doctoral Guidance Committee of [*first author*] at the faculty of Psychology and Educational Sciences of Vrije Universiteit Brussels, and by the Palestinian Ministry of Detainees and Ex-Detainees Affairs; the latter also permitted to family records and contact information of Palestinian detainees in Israeli prisons. Permission and informed written consent was also obtained from all participating mothers. On the day of the interviews, the researcher explained the purpose of the study saying that the study was trying to understand the impact of parental detention on children’s psychological wellbeing. Referral for mental health support was available to all participants, upon request. All questionnaires were completed anonymously, and data were fully anonymized.

The Doctoral Guidance Committee of [*first author*] at the faculty of Psychology and Educational Sciences of Vrije Universiteit Brussels reviewed the study design and in particular this method of consent.

### Study sample

Geographically, the POT are divided into three parts: the West Bank, East Jerusalem and Gaza strip. For this study, the area of the West Bank was chosen, given that 89% of all Palestinians detained in Israeli prisons are from this region. The West Bank is divided into 11 governorships, of which we randomly selected four.

From the Ministry of Detainees and Ex-Detainees Affairs, we received a list of all Palestinian detainees in Israeli prisons (for the four selected governorships: total detainees: n = 2,020 of which 550 married). From the group of married detainees, we randomly (every third name on the list) selected 35% to be included in our sample (n = 183 families). From these families, we selected families with children between 3 and 10 years old, and, when there was more than one child in this age range, we asked mothers to complete questionnaires about one of their 3- to 10-years old children. This lead to a final sample of 79 children (mean age = 7.7 years, SD = 1.7; 43 (55.8%) boys and 34 (44.2%) girls) (table 2). 29 (36.7%) children saw the arrest of their fathers.

**Table 2:** Socio-demographic characteristics of the children.

	Total group (n = 178)	Father imprisoned (n = 79)	Father not imprisoned (n = 99)	Χ^2^/F
Gender				.379
Male	97 (54.4%	43 (55.8%)	54 (55.5%)	
Female	79 (44.4%)	34 (44.2%)	45 (45.5%)	
Age (Mean, SD)	7.7 (1.7)	7.7 (1.7)	7.6 (1.7)	F = .558
Housing situation				12.3*
Urban	59 (33.1%)	20 (25.3%)	39 (39.4%)	
Rural	56 (31.5%)	24 (30.4%)	32 (32.3%)	
Refugee camp	63 (35.4%)	35 (44.3%)	28 (28.3%)	
Living situation				26.3***
With fathers’ family	49 (25.8%)	10 (12.7%)	36 (36.4%)	
With mothers’ family	35 (19.7%)	10 (12.7%)	25 (25.3%)	
Separated house	95 (53.4%)	58(73.4%)	37 (37.4%)	
Did see the detention process				n.a.
Yes	n.a.	29 (36.7%)	n.a.	
No	n.a.	49 (62.2%)	n.a.	

N(%);

*p<.05,

***p<0.001

The comparison group (children whose father was not in detention) was sampled from the same governorships: in cooperation with the Palestinian Ministry of Education and the Ministry of Health, we received a list of schools for girls (n = 190) and a list of health centers (n = 20). From this list, we randomly took each fifth school and each second health center, and asked all employees (mostly females) to participate to the research. Of the 1,300 employees, all teachers, social workers or nurses, about 1,100 were married, and 1,000 completed the questionnaires. Of those, 177 females had never been confronted with detention in their family and thus could be included in the comparison sample. In this group, 99 females had children aged 3–10 years old, and completed questionnaires about one of their children in this age range (if they had more than one) (n = 99; mean age = 7.6 years, SD = 1.7; 54 (54.5%) boys and 45 (45.5%) girls). Some differences between both groups are presented in table 2: children whose father is detained lived more in refugee camps and were more likely not to live with their extended family.

### Procedure

Data regarding the impact of fathers’ arrest and imprisonment on the psychological wellbeing of their children was collected by the use of two questionnaires that were filled in anonymously by the 178 mothers of the children between 3 and 10 years old, whose fathers were (n = 79) or were not (n = 99) in prison (S1 Data). The first author and a team of three female social workers trained in research methodology visited the families at home, informed them about the research and its aims, asked to complete an informed consent, and invited the mothers to complete the questionnaires anonymously. The researcher remained present to support the mothers if needed. The mothers whose husbands were never imprisoned were visited at their working place by one of the aforementioned researchers; the research aims were explained, the informed consent signed, and information was provided on how to complete the questionnaires. The researcher returned the next day to receive the completed questionnaires.

### Measures

Next to a socio-demographic questionnaire, investigating age, gender, living environment (urban, rural, refugee camp), housing situation (separated house, living in father’s family house or in mother’s family house) and, in case of, whether the child witnessed the arrest and detention process of his/her father, mothers were asked to complete two questionnaires about their child’s psychological wellbeing, both questionnaires already used in Palestinian context. Before data collection, the use of the questionnaires was discussed in a group of seven experts from Palestinian universities to verify their suitability for the research aims and population, and a pilot testing was done with a group of 15 Palestinian mothers with 3–10 years old children whose husbands were imprisoned. This sample was excluded from further analyses.

*UCLA-PTSD-Reaction Index* (UCLA-PTSD-RI) [30]: based on the DSM-IV criteria for PTSD, this questionnaire has versions for children, adolescents and parents, and has been widely used in research on PTSD in children and adolescents [31]. 22 items are scored on a Likert-scale from 0 (*never*) to 4 (*always*). For this study, we used the Arabic version [31], which is adapted to the Palestinian context. However, reliability analyses showed a low or even negative correlation of three items (item 14: ‘I think that some part of what happened is my fault’, item 15: ‘I have trouble remembering important parts of what happened’, and item 17: ‘I try to stay away from people places, or things that make me remember what happened’) with the total score. Therefore, these items were not used when calculating the total score. Total scores higher than 30 (pc 85 in our sample) were considered high scores (cut-off). Cronbach’s alpha without the omitted items was .90.

Strengths and Difficulties Questionnaire (SDQ) [32]: This questionnaire is widely used to assess the psychological wellbeing of children [32–33]. It contains 25 items to be scored on a Likert-scale from (0 to 2) respectively (not true, somewhat true, and certainly true); five subscales are distinguished (pro-social behavior, hyperactivity, emotional symptoms, conduct problems, and peer problems), as also a total difficulties score (sum of the 20 items from the four problems scales). In this study, we used the Arabic translation of the SDQ parent version, which has already been used in Palestine [34–35]. Reliability analyses showed that four items poorly correlated with the total problem score (item 7: ‘I usually do as I am told’, item 11: ‘I have one good friend or more’, item 14: ‘Other people may age generally like me’, and item 22: ‘I take things that are not mine’), which were therefore omitted from the calculation of the total score and subscale scores. Omitting these items led to a Cronbach’s alpha of .89 of the total problem score, and .86 and .67 for the emotional and hyperactivity subscales respectively. The conduct and peer problems subscales were not used as separate subscales, because of their low internal reliability. A total problem score higher than 20 (pc 85 in our sample) was considered a high score (cut-off score). For the subscales emotional problems and hyperactivity, scores higher than 7 (percentile 85) were considered high scores.

### Statistical analysis

Descriptive statistics, ANOVA and chi-square analyses were used to present sample’s demographic characteristics and prevalence of psychological problems. Logistic regression analyses (method: enter) were conducted to analyze possible differences between both groups (children with and without a father in detention) and to determine the variables impacting the psychological wellbeing of captives’ children (age, gender, housing situation, and living situation). Next, a second series of analyses were executed only in the group of children whose father is imprisoned to investigating the impact of witnessing the father’s arrest onto children’s mental health. Analyses were performed using SSPS (version 22).

## Results

### Psychological wellbeing

For about one quarter to one third of the young children whose fathers were in prison, mothers reported scores above the threshold, showing they had severe psychological problems (SDQ) and severe symptoms of posttraumatic stress (UCLA-PTSD-RI). These percentages are much higher than in the control group of children whose fathers were not detention (table 3). Logistic regression analyses confirm the large impact of father’s detention onto young children’s psychological wellbeing (table 4).

**Table 3:** Percentages of different groups with severe psychological problems (above threshold scores).

	Total group (n = 178)	Father imprisoned (n = 79)	Father not imprisoned (n = 99)	Chi-square
Total PTSD (>30)	24 (13.5%)	20 (25.3%)	4 (4.3%)	16.1***
SDQ				
Total difficulties score (>20)	27 (15.2%)	25 (31.6%)	2 (2.1%)	29.0
Emotional problems subscale (>7)	24 (13.5%)	20 (25.3%)	1 (1.0%)	16.2***
Hyperactivity subscale (>7)	27 (15.2%)	26 (32.9%)	4 (4.2%)	33.7***

N(%);

***p<0.001.

PTSD: posttraumatic stress disorder as measured by the USCL-PTSD Reaction Index Questionnaire. SDQ: Strengths and Difficulties Questionnaire.

**Table 4:** Logistic regression analyses examining the impact of father’s detention and sociodemographic variables on children’s psychological wellbeing (total group).

	Total PTSD		SDQ-Total difficulties		Emotional problems subscale		Hyperactivity subscale	
	Exp(B)	Wald	Exp(B)	Wald	Exp(B)	Wald	Exp(B)	Wald
Group (ref = not imprisoned)	-.085	13.308***	-.035	16.314***	-.095	13.615**	-.016	14.248***
Age	1.088	.015	-.767	3.058*	.990	.005	-.736	3.676*
Gender (ref = female)	1.093	.034	.677	.578	.874	.075	-.234	6.356**
Housing situation (ref = rural)		6.364*		1.638		2.172		.340
Urban	.557	.935	1.054	.004	1.008	.001	.670	.294
Refugee camp	-.188	6.306**	.515	1.085	.448	1.677	.941	.009
Living situation (ref = separated house)		3.381		4.565		2.521		2.923
With father’s family	.278	2.123	-.141	2.906*	.769	.125	.354	1.189
With mother’s family	1.617	.538	2.054	1.096	2.405	1.859	2.484	1.304

Exp(B);

*p<.05,

**p<.01,

***p<.001.

Total PTSD: R^2^ = .282; SDQ total difficulties: R^2^ = .389, SDQ emotional problems: R^2^ = .220, SDQ hyperactivity: R^2^ = .468. PTSD: posttraumatic stress disorder as measured by the USCL-PTSD Reaction Index Questionnaire; SDQ: Strengths and Difficulties Questionnaire.

Above, younger children established slightly more total difficulties (SDQ) and more problems of hyperactivity (SDQ), the latter also more frequently reported in boys. Children living in rural areas had higher risks to developing symptoms of PTSD, compared to children living in refugee camps. The living arrangement only had a small influence on children’s psychological problems, with children living in separated houses (not with the extended family) having slightly higher risk to developing psychological problems (total SDQ difficulties scores) compared to those living in the house of their father’s family.

### Witnessing father’s arrest and detention process

Examining only the group of children whose parents were detained, we found that those who watched the arrest and detention process of their father scored largely higher on all mental health problems (table 5). Above, boys established more hyperactive problems (SDQ) compared to girls, and children living in rural areas had higher risks to developing symptoms of PTSD compared to children living in refugee camps. Children living together with their mother’s family showed slightly more problems of hyperactivity (SDQ) then those living only with their mother in separated housing. No influence of age was found on the different mental health scales.

**Table 5:** Logistic regression analyses examining the impact of viewing father’s detention process and sociodemographic variables on the psychological wellbeing of children whose father is imprisoned.

	Total PTSD		SDQ-Total difficulties		Emotional problems subscale		Hyperactivity subscale	
	Exp(B)	Wald	Exp(B)	Wald	Exp(B)	Wald	Exp(B)	Wald
Viewed detention (ref = did not view)	-.209	5.705**	-.187	7.058**	-.199	6.090**	-.308	3.797*
Age	.879	.484	.764	2.466	.884	.487	.812	1.577
Gender (ref = female)	1.197	.077	.855	.044	1.744	.813	-.237	5.708**
Housing situation (ref = rural)		4.902*		.690		2.464		1.166
Urban	.389	1.281	1.569	.331	1.457	.234	.901	.017
Refugee camp	-.159	4.891*	.893	.023	.444	1.068	1.803	.703
Living situation (ref = separated house)		4.085		3.227		1.458		3.342
With father’s family	.151	2.229	.252	1.283	1.109	.000	.620	.236
With mother’s family	2.968	1.428	3.024	1.661	2.967	1.434	4.667	2.936*

Exp(B);

*p<.05,

**p<.01.

Total PTSD: R^2^ = .345; SDQ total difficulties: R^2^ = .280, SDQ emotional problems: R^2^ = .271, SDQ hyperactivity: R^2^ = .261. PTSD: posttraumatic stress disorder as measured by the USCL-PTSD Reaction Index Questionnaire; SDQ: Strengths and Difficulties Questionnaire.

## Discussion

The aim of the study was, firstly, to investigate the impact of the arrest and imprisonment of the father on the psychological wellbeing of young Palestinian children, and, secondly, the psychological consequences of witnessing the capturing of the fathers.

Imprisonment of the father in contexts of protracted armed conflict largely impacts the psychological wellbeing of children: 14.2% of the Palestinian children whose father is imprisoned showed serious symptoms of PTSD (UCLA-PTSD-RI), this is twelve times higher than children who are not confronted with parental imprisonment. This finding relates to other studies [9,25,27, 36–38]. Besides, 15.4% of arrested father’s children reported psychological and behavioral problems according to the SDQ, with a prevalence of 28 times higher than children without experiences of detention in their family. Of the captive’s children, 13.7% got SDQ scores indicating emotional problems, ten times higher than in the group of children without parental imprisonment. The aforementioned findings are in line with other studies indicating higher prevalence of PTSD symptoms [3,6,8,39] and/or mental health problems [4,6,25,23,24,36,37,40,41] in situations of parental imprisonment.

Given that arrest of Palestinian men often happens at checkpoints on the street, and frequently during the night while surrounding the house, knocking with guns on the doors, sometimes blowing up doors or launching tear gas and sound bombs, many Palestinian children witness their father’s arrest, being hit, handcuffed and blindfolded. Witnessing father’s arrest significantly increased the prevalence of PTSD symptoms in the young children involved in this study. PTSD occurred 5 times more in the group of children who saw their father’s detention, compared to those who did not see it. And about one third of the children who witnessed their father’s arrest showed SDQ psychological and behavioral difficulties, five times more than those children who did not watch the detention process.

Children’s gender was not significantly related to PTSD, behavior difficulties and emotional problems, which is in line with some studies [15,16], while contrasting other studies, the latter showing that girls established higher mental health problems than boys, especially concerning PTSD and emotional problems [2–4,8,10,11,17,24,37,39]. Only for the SDQ hyperactivity subscale, boys showed higher scores, which is also confirming earlier studies [39].

Considering age, the study findings indicate that the younger the child is, the more psychological and behavioral problems (SDQ) (s)he might have as a consequence of the imprisonment of the father. This finding might be consistent with previous studies, suggesting that younger children are more likely to being influenced by their parents’ responses to traumatic or stressful events than older children [6,10,11,39,42], however, no significant differences in PTSD were found related to age. Above, age differences did not influence the relationship between witnessing father’s arrest and the prevalence of PTSD, behavior and emotional problems.

The study indicated some influence of the living environment on the prevalence of PTSD: Children living in villages showed three to five times higher risk on having PTSD than those living in urban environments or refugee camps. This can be interpreted by two Palestinian socio-demographic facts: First, most of the extended families in Palestine still live in villages, and, secondly, those villages are located in C-areas—these are areas that are more likely to being affecting by ongoing conflicts with soldiers and settlers than other areas in the POT. Besides, children living in their father’s family house tended to have more SDQ psychological and behavioral problems. This could be due to higher levels of interference in the children’s personal lives by members of the father’s extended family because of his absence. As such, children’s privacy and personal spaces to experiment become more limited, which might hamper their psychological wellbeing. Another possible hypothesis here is that the intensified presence of extended family members increases (explicit or implicit) communication about the situation of the father, and the child's witnessing of anxiety and distress in these caregiving figures, which then lead to increased stress in the child. Above, this particular living situation might also impact the mental health of the mothers, which then in turn may be associated importantly with increased distress in the children, as we know out of several other studies, including some in the war-affected area of the Gaza Strip [43–45].

In sum, the findings of this study point to the seriousness of the psychological consequences onto children by the frequently occuring practices of arrest and long-term imprisonment of Palestinian fathers in the Palestinian Occupied Territories, more specifically in the West Bank.

### Implications

Given the large impact of parental detention on the psychological wellbeing of young children, there is a huge need for more and in-depth support for children whose father (or another family member) is detained. This support could, firstly, be expected from governmental institutions by, amongst others, installing outreaching teams to can provide psychological support at home, for both mothers and children. However, given the involvement of governmental organisations within the protracted political conflict, also non-governmental organizations should take up their roles, through providing of in-depth psychological and social support to family members of detainees, in particular children, both at home as in accessible services. Besides these direct support initiatives, also schools can be used an entry point to reach these children, for example through activating the role of educational counselors who could give support to these children and their contexts, and also refer children to more in-depth mental health care if needed. Further, creating more and structured possibilities to ensure communication between detained fathers and their children could also lower the impact of the detention onto children’s psychological wellbeing. However, clearly, the main implication of the study is that, given the wide-scale negative impact of these phenomena, all involved parties and actors should largely urge to ending wars and political conflicts as fast as possible.

### Limitations

We are aware of at least four main limitations in conducting this study. First, data were collected only from the mothers of children aged between 3 and 10 years old. Taking into consideration that mothers’ observation of their children’s behavior can be influenced by their own experiences of being exposed to stressful and traumatic experiences, and considering the findings of Wolmer and Cohen [28], stating that the effects on parents resulting from traumatic stressors may affect the child’s well-being more than the experience of the trauma by the child him/herself, future research requires the collection of data from also other and different sources, such as teachers and the children themselves. Secondly, there were some differences in socio-demographic characteristics between both groups, and other differences, such as biological or environmental characteristics, that were not measured in this study might also have impacted the study findings. Thirdly, there might also have been differences in traumatic exposure between both groups, which could have impacted the children’s wellbeing. Further, participants may have altered their ways of participation, given that they were informed on beforehand about the particular aims of this study. A final limitation of this study is the fact that only one part of the Palestinian Occupied Territories (POT) was involved. Consequently, future research could be strengthened by including the other two parts of the POT.

## Supporting Information

S1 Data Raw data. This database contains the raw data onto which this paper is based.
(PDF)
