# Supplementary material for: The Impact of Parental Detention on the Psychological Wellbeing of Palestinian Children
Source: PLoS One. 2015 Jul 17;10(7):e0133347. doi: 10.1371/journal.pone.0133347 (PMC4506043; doi:10.1371/journal.pone.0133347)
Supplement: S1 Data — This database contains the raw data onto which this paper is based. (PDF) [file pone.0133347.s001.pdf]

ID	group	Gender	age	adress	viewdetention	accommodation	educationf	educationm	educationff	a1	a2	a3	a4	a5	a6	a7
1,00	test	male	6,00	refugee camp	No	separated house	secondary	diploma	diploma	Maybe	No	No	Much	No	No	No
2,00	test	female	7,00	refugee camp	No	separated house	high education	diploma	high education	No	No	No	Maybe	No	No	No
3,00	test	female	9,00	city	No	separated house	elementary	secondary	elementary	Maybe	No	No	Much	Maybe	No	Litlle
4,00	test	female	10,00	refugee camp	No	separated house	high education	high education	high education	No	No	No	Maybe	No	No	No
5,00	test	male	5,00	refugee camp	No	separated house	elementary	elementary	elementary	Maybe	Much	No	Maybe	No	No	Very much
6,00	test	female	7,00	refugee camp	No	separated house	high education	diploma	high education	No	No	No	Much	Maybe	No	No
7,00	test	female	8,00	refugee camp	yes	separated house	diploma	diploma	diploma	Much	Much	Much	Much	Much	No	No
8,00	test	male	9,00	refugee camp	yes	separated house	secondary	diploma	diploma	No	Maybe	Maybe	Much	No	No	No
9,00	test	male	10,00	city	No	separated house	primary	secondary	secondary	No	No	No	Maybe	No	No	Maybe
10,00	test	male	10,00	vellage	No	separated house	diploma	high education	high education	Maybe	Maybe	Litlle	Much	Maybe	Maybe	Maybe
11,00	test	999,00	7,00	refugee camp	No	with mothers family	primary	secondary	secondary	Litlle	Maybe	Litlle	Much	Maybe	No	No
12,00	test	male	8,00	refugee camp	No	separated house	secondary	diploma	diploma	Much	No	Litlle	Very much	No	No	No
13,00	test	female	5,00	refugee camp	No	separated house	high education	secondary	high education	Much	Much	No	Much	Maybe	No	No
14,00	test	female	5,00	vellage	yes	separated house	secondary	high education	high education	Maybe	Litlle	Litlle	Litlle	No	Litlle	Maybe
15,00	test	female	6,00	vellage	No	with mothers family	secondary	secondary	secondary	Maybe	Very much	No	Much	Litlle	Litlle	Litlle
16,00	test	male	7,00	city	No	separated house	secondary	elementary	secondary	No	Much	Maybe	Much	No	No	No
17,00	test	male	6,00	refugee camp	No	separated house	diploma	elementary	diploma	No	No	Litlle	Maybe	Maybe	No	No
18,00	test	male	5,00	refugee camp	No	with mothers family	elementary	elementary	elementary	No	No	No	Much	No	No	No
19,00	test	male	9,00	refugee camp	No	separated house	high education	high education	high education	Litlle	Litlle	No	Maybe	Maybe	Litlle	No
20,00	test	male	10,00	refugee camp	yes	separated house	high education	high education	high education	No	No	No	Maybe	No	No	No
21,00	test	female	10,00	refugee camp	yes	separated house	diploma	secondary	diploma	No	No	No	Very much	No	No	No
22,00	test	male	5,00	refugee camp	yes	separated house	secondary	elementary	secondary	Litlle	Maybe	Maybe	Much	No	No	Maybe
23,00	test	999,00	7,00	refugee camp	yes	separated house	high education	high education	high education	Maybe	Litlle	No	No	No	No	No
24,00	test	male	8,00	refugee camp	No	separated house	diploma	diploma	diploma	Maybe	No	No	Much	No	No	No
25,00	test	female	8,00	vellage	No	separated house	diploma	diploma	diploma	Maybe	Much	Much	Maybe	No	No	No
26,00	test	male	6,00	refugee camp	No	separated house	high education	secondary	high education	No	Litlle	No	No	Litlle	No	No
27,00	test	female	8,00	refugee camp	No	separated house	elementary	high education	high education	Maybe	Maybe	No	Much	Maybe	Maybe	Maybe
28,00	test	male	9,00	refugee camp	No	separated house	elementary	high education	high education	No	No	No	Much	Litlle	No	No
29,00	test	female	10,00	refugee camp	No	with mothers family	elementary	diploma	diploma	Litlle	No	No	No	Litlle	No	Litlle
30,00	test	male	10,00	refugee camp	yes	with fathers family	secondary	elementary	secondary	No	No	Litlle	No	No	No	No
31,00	test	female	10,00	vellage	yes	separated house	elementary	diploma	diploma	Very much	Much	Much	Much	Much	Much	Litlle
32,00	test	male	9,00	vellage	yes	separated house	elementary	secondary	secondary	Much	Much	Much	Much	Much	Much	Maybe
33,00	test	female	9,00	vellage	yes	separated house	elementary	secondary	secondary	Much	Very much	Much	Much	Much	Much	Much
34,00	test	female	9,00	refugee camp	No	separated house	high education	high education	high education	Maybe	Much	No	No	No	No	No
35,00	test	female	7,00	refugee camp	No	separated house	diploma	high education	high education	Much	Much	No	Much	No	No	No
36,00	test	male	6,00	refugee camp	No	separated house	secondary	high education	high education	Maybe	Much	Much	Much	No	No	No
37,00	test	female	5,00	refugee camp	No	separated house	secondary	high education	high education	No	Maybe	No	Maybe	No	No	Maybe
38,00	test	female	8,00	refugee camp	No	separated house	secondary	elementary	secondary	Maybe	Much	Maybe	Much	Maybe	Maybe	Maybe
39,00	test	male	9,00	vellage	No	separated house	high education	high education	high education	Maybe	No	No	Litlle	No	No	No
40,00	test	male	10,00	city	No	separated house	diploma	high education	high education	Much	Much	No	Very much	No	No	No
41,00	test	female	10,00	city	No	with mothers family	secondary	high education	high education	Maybe	Much	No	Much	No	No	No
42,00	test	female	5,00	city	yes	separated house	high education	high education	high education	No	No	Maybe	Litlle	No	Much	No
43,00	test	male	6,00	city	No	separated house	elementary	secondary	secondary	Litlle	No	No	Litlle	Litlle	No	Maybe
44,00	test	male	7,00	city	No	separated house	elementary	high education	high education	No	Maybe	No	Litlle	Litlle	No	No
45,00	test	male	8,00	refugee camp	No	separated house	high education	diploma	high education	No	No	No	No	Litlle	No	No
46,00	test	female	9,00	city	yes	separated house	high education	diploma	high education	Very much	Maybe	Maybe	Much	Maybe	No	No
47,00	test	male	10,00	city	yes	999,00	high education	high education	high education	No	Maybe	No	Maybe	No	No	Litlle
48,00	test	female	10,00	city	No	separated house	elementary	secondary	secondary	Litlle	Maybe	No	Maybe	Maybe	No	No

49,00	test	female	9,00	city	yes	separated house	elementary	secondary	secondary	Maybe	Maybe	Litlle	Much	Maybe	Litlle	No
50,00	test	male	8,00	city	yes	separated house	high education	diploma	high education	Maybe	Maybe	Maybe	Very much	Maybe	Litlle	No
51,00	test	male	7,00	city	yes	separated house	primary	elementary	diploma	Maybe	Maybe	Much	Much	Maybe	Litlle	Much
52,00	test	female	6,00	city	No	with mothers family	secondary	diploma	diploma	Much	Maybe	Litlle	Much	Maybe	No	Much
53,00	test	male	5,00	city	No	with mothers family	elementary	elementary	elementary	Maybe	Much	Much	Very much	Much	Maybe	No
54,00	test	female	7,00	city	No	separated house	secondary	elementary	secondary	Maybe	Maybe	No	Maybe	No	No	No
55,00	test	male	8,00	city	yes	with mothers family	primary	primary	primary	Much	Much	Much	Much	Much	Much	No
56,00	test	female	6,00	city	yes	with mothers family	high education	high education	high education	Much	Much	Much	Maybe	Much	Much	No
57,00	test	male	5,00	vellage	No	with fathers family	elementary	elementary	elementary	Maybe	No	No	Maybe	No	No	No
58,00	test	male	9,00	vellage	yes	separated house	secondary	secondary	secondary	Much	Much	Maybe	Much	Maybe	No	Much
59,00	test	male	10,00	vellage	No	separated house	secondary	elementary	secondary	Maybe	Much	Maybe	Much	No	No	No
60,00	test	male	9,00	vellage	No	separated house	secondary	elementary	secondary	No	Much	Very much	Much	Litlle	No	No
61,00	test	male	8,00	vellage	yes	separated house	elementary	high education	high education	Litlle	Litlle	No	No	No	Maybe	No
62,00	test	female	7,00	vellage	999,00	with fathers family	high education	high education	high education	Much	Very much	Maybe	Much	Maybe	Maybe	No
63,00	test	male	5,00	vellage	yes	with fathers family	high education	diploma	high education	No	Very much	Maybe	Much	Maybe	Maybe	Much
64,00	test	male	7,00	vellage	No	with fathers family	high education	high education	high education	Much	Very much	No	Much	Maybe	Maybe	No
65,00	test	female	6,00	vellage	No	with fathers family	high education	diploma	high education	Maybe	Maybe	No	Maybe	Maybe	No	No
66,00	test	male	8,00	vellage	No	with fathers family	secondary	diploma	diploma	No	No	No	No	No	No	No
67,00	test	female	10,00	refugee camp	No	separated house	diploma	diploma	diploma	No	Maybe	No	Litlle	No	No	No
68,00	test	male	7,00	refugee camp	No	with mothers family	secondary	elementary	secondary	No	No	No	Much	Much	No	No
69,00	test	male	8,00	vellage	yes	separated house	high education	high education	high education	Maybe	Much	Much	Much	Very much	Maybe	Maybe
70,00	test	male	6,00	refugee camp	No	separated house	high education	high education	high education	Maybe	Maybe	No	No	No	No	No
71,00	test	female	10,00	vellage	yes	with fathers family	diploma	high education	high education	Much	Much	Maybe	Much	Much	Maybe	No
72,00	test	male	5,00	vellage	yes	separated house	secondary	secondary	secondary	Maybe	Much	Much	Much	Much	Very much	Maybe
73,00	test	male	8,00	vellage	yes	separated house	elementary	high education	high education	Much	Very much	Very much	Very much	Very much	Much	Very much
74,00	test	male	9,00	vellage	yes	separated house	elementary	secondary	secondary	Much	No	Maybe	Much	No	Very much	No
75,00	test	female	10,00	vellage	No	with fathers family	elementary	diploma	diploma	Maybe	No	No	Maybe	No	No	No
76,00	test	female	9,00	refugee camp	No	separated house	secondary	high education	high education	No	No	No	Maybe	No	No	No
77,00	test	female	6,00	refugee camp	No	with fathers family	high education	diploma	high education	Much	Much	Much	Much	Maybe	No	No
78,00	test	female	7,00	city	yes	separated house	high education	diploma	high education	Much	Litlle	No	Litlle	Litlle	No	No
79,00	test	male	8,00	refugee camp	yes	separated house	secondary	diploma	diploma	No	Much	Much	Much	No	No	No
80,00	control	male	8,00	city	nvt	with fathers family	high education	high education	high education	Litlle	Litlle	Litlle	No	No	No	No
81,00	control	female	6,00	refugee camp	nvt	with fathers family	high education	high education	high education	Litlle	Litlle	Litlle	No	No	No	No
82,00	control	female	5,00	city	nvt	separated house	high education	high education	high education	Litlle	Litlle	Litlle	Litlle	Litlle	No	No
83,00	control	female	7,00	city	nvt	separated house	high education	high education	high education	Litlle	Litlle	Litlle	Litlle	Litlle	No	No
84,00	control	female	8,00	city	nvt	separated house	high education	high education	high education	Litlle	Litlle	Litlle	Litlle	Litlle	No	No
85,00	control	female	10,00	city	nvt	separated house	high education	high education	high education	Litlle	Litlle	Litlle	Litlle	Litlle	No	No
86,00	control	male	7,00	vellage	nvt	with mothers family	high education	high education	high education	Maybe	Maybe	Maybe	Litlle	Litlle	Litlle	Litlle
87,00	control	female	6,00	city	nvt	separated house	high education	high education	high education	Litlle	Litlle	Litlle	Litlle	Litlle	No	No
88,00	control	male	8,00	vellage	nvt	with mothers family	high education	high education	high education	Maybe	Maybe	Maybe	Litlle	Litlle	Litlle	Litlle
89,00	control	female	8,00	refugee camp	nvt	with fathers family	high education	high education	high education	Litlle	Litlle	Litlle	No	No	No	No
90,00	control	female	7,00	refugee camp	nvt	with fathers family	high education	high education	high education	Litlle	Litlle	Litlle	No	No	No	No
91,00	control	female	9,00	refugee camp	nvt	with fathers family	high education	high education	high education	Litlle	Litlle	Litlle	No	No	No	No
92,00	control	female	10,00	refugee camp	nvt	with fathers family	high education	high education	high education	Litlle	Litlle	Litlle	No	No	No	No
93,00	control	male	8,00	vellage	nvt	with fathers family	high education	high education	high education	No	No	No	No	No	No	No
94,00	control	male	7,00	city	nvt	separated house	high education	high education	high education	No	Maybe	Litlle	Maybe	No	No	No
95,00	control	male	3,00	vellage	nvt	separated house	high education	high education	high education	Litlle	Maybe	Maybe	Maybe	Maybe	Maybe	Maybe
96,00	control	male	4,00	refugee camp	nvt	with fathers family	high education	high education	high education	No	Maybe	No	No	No	Maybe	No
97,00	control	male	9,00	city	nvt	separated house	high education	high education	high education	Maybe	Maybe	Maybe	Litlle	Litlle	Litlle	Litlle

98,00	control	male	8,00	vellage	nvt	with mothers family	high education	high education	high education	Maybe	Maybe	Maybe	Litlle	Litlle	Litlle	Litlle
99,00	control	male	7,00	vellage	nvt	with mothers family	high education	high education	high education	Maybe	Maybe	Maybe	Litlle	Litlle	Litlle	Litlle
100,00	control	male	9,00	vellage	nvt	separated house	high education	high education	high education	No	No	No	No	No	No	No
101,00	control	female	8,00	vellage	nvt	with mothers family	high education	high education	high education	No	No	No	No	No	No	No
102,00	control	female	8,00	vellage	nvt	with mothers family	high education	high education	high education	No	No	No	No	No	No	No
103,00	control	female	7,00	vellage	nvt	with mothers family	high education	high education	high education	No	No	No	No	No	Litlle	No
104,00	control	male	10,00	vellage	nvt	with fathers family	high education	high education	high education	No	No	No	No	No	No	No
105,00	control	female	10,00	city	nvt	with mothers family	high education	high education	high education	No	No	No	No	No	No	No
106,00	control	female	5,00	city	nvt	separated house	high education	high education	high education	Litlle	Litlle	Litlle	Litlle	Litlle	No	No
107,00	control	male	9,00	vellage	nvt	with mothers family	high education	high education	high education	Maybe	Maybe	Maybe	Litlle	Litlle	Litlle	Litlle
108,00	control	male	4,00	refugee camp	nvt	separated house	high education	high education	high education	Litlle	Litlle	Litlle	Litlle	No	No	No
109,00	control	female	10,00	vellage	nvt	separated house	high education	high education	high education	No	Litlle	Maybe	Litlle	No	No	No
110,00	control	male	8,00	city	nvt	with mothers family	high education	high education	high education	No	No	No	No	No	No	No
111,00	control	female	10,00	city	nvt	999,00	high education	high education	high education	No	No	No	No	No	No	No
112,00	control	male	4,00	refugee camp	nvt	separated house	high education	high education	high education	No	No	No	No	No	No	No
113,00	control	male	10,00	city	nvt	separated house	high education	high education	high education	No	No	No	No	No	No	No
114,00	control	female	8,00	city	nvt	with mothers family	high education	high education	high education	Maybe	Maybe	Maybe	Maybe	Maybe	Maybe	Maybe
115,00	control	female	7,00	vellage	nvt	separated house	high education	high education	high education	No	No	No	No	No	No	No
116,00	control	female	6,00	city	nvt	with fathers family	high education	high education	high education	No	No	No	No	No	No	No
117,00	control	female	7,00	refugee camp	nvt	with mothers family	high education	high education	high education	No	No	No	No	No	Litlle	No
118,00	control	male	10,00	vellage	nvt	with mothers family	high education	high education	high education	No	Maybe	Litlle	Litlle	Very much	Much	Litlle
119,00	control	male	6,00	city	nvt	with fathers family	high education	high education	high education	No	No	No	No	No	No	No
120,00	control	male	6,00	vellage	nvt	with mothers family	high education	high education	high education	No	No	No	No	No	No	Litlle
121,00	control	female	6,00	city	nvt	separated house	high education	high education	high education	No	No	No	No	No	No	Litlle
122,00	control	male	6,00	city	nvt	with fathers family	high education	high education	high education	No	Litlle	Litlle	Maybe	Litlle	Litlle	No
123,00	control	male	9,00	city	nvt	with fathers family	high education	high education	high education	No	Litlle	Litlle	Maybe	Litlle	Litlle	No
124,00	control	male	10,00	refugee camp	nvt	separated house	high education	high education	high education	Litlle	Litlle	Litlle	Litlle	Litlle	No	No
125,00	control	female	6,00	vellage	nvt	separated house	high education	high education	high education	No	Litlle	Litlle	No	No	Litlle	No
126,00	control	male	6,00	refugee camp	nvt	separated house	high education	high education	high education	Litlle	Litlle	Litlle	Litlle	Litlle	No	No
127,00	control	male	7,00	city	nvt	with fathers family	high education	high education	high education	Litlle	Litlle	Litlle	No	No	No	No
128,00	control	male	8,00	city	nvt	with fathers family	high education	high education	high education	No	Litlle	Litlle	Maybe	Litlle	Litlle	No
129,00	control	female	6,00	vellage	nvt	with fathers family	high education	high education	high education	No	Litlle	Maybe	Litlle	Litlle	No	No
130,00	control	female	8,00	vellage	nvt	with mothers family	high education	high education	high education	No	Litlle	Maybe	Litlle	Litlle	No	No
131,00	control	female	6,00	vellage	nvt	with fathers family	high education	high education	high education	No	Litlle	Maybe	Litlle	Litlle	No	No
132,00	control	male	9,00	city	nvt	with fathers family	high education	high education	high education	Litlle	Litlle	Litlle	No	No	No	No
133,00	control	male	8,00	refugee camp	nvt	separated house	high education	high education	high education	Litlle	Litlle	Litlle	Litlle	Litlle	No	No
134,00	control	male	10,00	city	nvt	with fathers family	high education	high education	high education	Litlle	Litlle	Litlle	No	No	No	No
135,00	control	male	6,00	city	nvt	with fathers family	high education	high education	high education	Litlle	Litlle	Litlle	No	No	No	No
136,00	control	male	7,00	city	nvt	with fathers family	high education	high education	high education	Litlle	Litlle	Litlle	No	No	No	No
137,00	control	male	8,00	city	nvt	with fathers family	high education	high education	high education	Litlle	Litlle	Litlle	No	No	No	No
138,00	control	male	6,00	city	nvt	with fathers family	high education	high education	high education	Litlle	Litlle	Litlle	No	No	No	No
139,00	control	male	6,00	city	nvt	with fathers family	high education	high education	high education	Litlle	Litlle	Litlle	No	No	No	No
140,00	control	female	5,00	city	nvt	separated house	high education	high education	high education	Litlle	Litlle	Litlle	Litlle	Litlle	No	No
141,00	control	female	5,00	city	nvt	separated house	high education	high education	high education	Litlle	Litlle	Litlle	Litlle	Litlle	No	No
142,00	control	female	6,00	vellage	nvt	with mothers family	high education	high education	high education	No	Litlle	Maybe	Litlle	Litlle	No	No
143,00	control	male	7,00	refugee camp	nvt	separated house	high education	high education	high education	Litlle	Litlle	Litlle	Litlle	Litlle	No	No
144,00	control	female	7,00	vellage	nvt	with mothers family	high education	high education	high education	No	Litlle	Maybe	Litlle	Litlle	No	No
145,00	control	female	6,00	vellage	nvt	with mothers family	high education	high education	high education	No	Litlle	Maybe	Litlle	Litlle	No	No
146,00	control	male	8,00	refugee camp	nvt	separated house	high education	high education	high education	Litlle	Litlle	Litlle	Litlle	Litlle	No	No

147,00	control	female	7,00	vellage	nvt	with mothers family	high education	high education	high education	No	Litlle	Maybe	Litlle	Litlle	No	No
148,00	control	female	10,00	vellage	nvt	with mothers family	high education	high education	high education	No	Litlle	Maybe	Litlle	Litlle	No	No
149,00	control	female	7,00	vellage	nvt	with mothers family	high education	high education	high education	No	Litlle	Maybe	Litlle	Litlle	No	No
150,00	control	male	10,00	refugee camp	nvt	separated house	high education	high education	high education	Litlle	Litlle	Litlle	Litlle	Litlle	No	No
151,00	control	male	7,00	refugee camp	nvt	separated house	high education	high education	high education	Litlle	Litlle	Litlle	Litlle	Litlle	No	No
152,00	control	male	6,00	refugee camp	nvt	separated house	high education	high education	high education	Litlle	Litlle	Litlle	Litlle	Litlle	No	No
153,00	control	male	9,00	refugee camp	nvt	separated house	high education	high education	high education	Litlle	Litlle	Litlle	Litlle	Litlle	No	No
154,00	control	male	9,00	city	nvt	separated house	high education	high education	high education	999	999	999	999	999	999	999
155,00	control	male	8,00	city	nvt	separated house	high education	high education	high education	999	999	999	999	999	999	999
156,00	control	male	7,00	city	nvt	separated house	high education	high education	high education	No	Litlle	Litlle	Maybe	Litlle	Litlle	No
157,00	control	male	8,00	city	nvt	separated house	high education	high education	high education	No	Litlle	Litlle	Maybe	Litlle	Litlle	No
158,00	control	male	10,00	city	nvt	separated house	high education	high education	high education	No	Litlle	Litlle	Maybe	Litlle	Litlle	No
159,00	control	male	7,00	city	nvt	separated house	high education	high education	high education	No	Litlle	Litlle	Maybe	Litlle	Litlle	No
160,00	control	male	9,00	city	nvt	with fathers family	high education	high education	high education	No	Litlle	Litlle	Maybe	Litlle	Litlle	No
161,00	control	female	8,00	vellage	nvt	separated house	high education	high education	high education	Maybe	Maybe	Maybe	Maybe	Maybe	Maybe	Maybe
162,00	control	male	9,00	refugee camp	nvt	with fathers family	high education	high education	high education	No	No	No	No	No	No	No
163,00	control	female	8,00	refugee camp	nvt	with fathers family	high education	high education	high education	No	No	No	No	No	No	No
164,00	control	female	7,00	city	nvt	with fathers family	high education	high education	high education	No	No	No	Litlle	No	Litlle	Maybe
165,00	control	female	10,00	refugee camp	nvt	with fathers family	high education	high education	high education	No	No	No	Litlle	No	Litlle	Maybe
166,00	control	female	10,00	refugee camp	nvt	with fathers family	high education	high education	high education	No	Litlle	Maybe	Litlle	No	No	No
167,00	control	male	7,00	refugee camp	nvt	separated house	high education	high education	high education	No	No	No	No	No	No	Litlle
168,00	control	male	10,00	refugee camp	nvt	with fathers family	high education	high education	high education	No	Much	Maybe	Litlle	Very much	Much	Litlle
169,00	control	female	9,00	vellage	nvt	with fathers family	high education	high education	high education	No	No	No	No	No	No	No
170,00	control	male	9,00	vellage	nvt	with mothers family	high education	high education	high education	Maybe	Maybe	Maybe	Litlle	Litlle	Litlle	Litlle
171,00	control	male	9,00	vellage	nvt	with mothers family	high education	high education	high education	Maybe	Maybe	Maybe	Litlle	Litlle	Litlle	Litlle
172,00	control	female	8,00	refugee camp	nvt	with fathers family	high education	high education	high education	Litlle	Litlle	Litlle	No	No	No	No
173,00	control	female	8,00	refugee camp	nvt	with fathers family	high education	high education	high education	Litlle	Litlle	Litlle	No	No	No	No
174,00	control	male	9,00	vellage	nvt	with mothers family	high education	high education	high education	Maybe	Maybe	Maybe	Litlle	Litlle	Litlle	Litlle
175,00	control	male	8,00	vellage	nvt	with mothers family	high education	high education	high education	Maybe	Maybe	Maybe	Litlle	Litlle	Litlle	Litlle
176,00	control	female	5,00	city	nvt	separated house	high education	high education	high education	Litlle	Litlle	Litlle	Litlle	Litlle	No	No
177,00	control	female	8,00	refugee camp	nvt	with fathers family	high education	high education	high education	Litlle	Litlle	Litlle	No	No	No	No
178,00	control	female	7,00	refugee camp	nvt	with fathers family	high education	high education	high education	Litlle	Litlle	Litlle	No	No	No	No

a8	a9	a10	a11	a12	a13	a14	a15	a16	a17	a18	a19	a20	a21	a22	b1	b2	b3
No	No	Much	No	No	No	No	No	No	No	No	No	Maybe	No	No	Surelly true	Not true	moderately true
No	Maybe	No	No	No	Maybe	No	No	Much	No	No	No	Maybe	No	No	moderately true	Not true	Not true
Litlle	Much	Much	No	Much	Maybe	No	No	Maybe	Much	Maybe	Litlle	Much	Maybe	Much	Surelly true	moderately true	Surelly true
No	No	No	No	Maybe	No	No	Maybe	No	No	No	Maybe	Very much	Very much	Much	Surelly true	Surelly true	Not true
Maybe	Litlle	No	No	Maybe	Maybe	Maybe	No	Maybe	No	No	No	No	No	No	moderately true	Surelly true	Not true
No	No	No	No	Much	No	No	No	No	No	No	No	Much	No	No	Surelly true	Surelly true	Surelly true
No	No	Maybe	Maybe	Much	Litlle	No	No	No	Maybe	No	No	Much	No	Maybe	Surelly true	Surelly true	Not true
No	Maybe	No	Litlle	Much	Maybe	No	No	No	Much	Much	No	No	No	Litlle	Surelly true	Surelly true	Not true
Much	Very much	Much	Maybe	Maybe	No	No	No	No	No	Maybe	No	Very much	Maybe	Litlle	moderately true	Not true	Not true
Maybe	Maybe	Maybe	Maybe	Maybe	No	No	Litlle	Much	999	Maybe	Maybe	Maybe	Maybe	Maybe	moderately true	moderately true	moderately true
No	Much	No	No	Maybe	Litlle	No	Much	Very much	Much	Litlle	No	Much	Maybe	Litlle	Not true	Surelly true	Not true
No	Very much	Much	Maybe	No	Very much	No	No	No	No	No	Maybe	No	No	Much	Not true	Surelly true	Not true
No	No	Litlle	No	Much	No	No	No	No	No	No	No	No	No	Maybe	Surelly true	Surelly true	Not true
Much	Much	Maybe	Maybe	Maybe	Litlle	No	Litlle	No	Much	Maybe	No	Very much	Maybe	Maybe	Surelly true	Surelly true	moderately true
No	No	Maybe	No	Much	Litlle	No	Maybe	Maybe	No	Maybe	Maybe	Maybe	No	Maybe	Surelly true	Not true	Not true
No	Maybe	Maybe	No	Maybe	No	No	Much	No	No	Litlle	No	Very much	No	Maybe	moderately true	Surelly true	Not true
No	No	Very much	No	No	No	No	No	No	No	No	No	Maybe	No	No	Surelly true	Surelly true	Not true
No	Much	Litlle	Much	No	No	No	Maybe	Much	Very much	Much	No	No	Litlle	No	Surelly true	Not true	Not true
No	Maybe	No	No	No	No	No	No	Maybe	No	No	No	Very much	No	No	Surelly true	Surelly true	moderately true
No	No	No	No	No	No	No	Much	Litlle	Much	Litlle	No	Litlle	No	No	Surelly true	Surelly true	Not true
No	Litlle	No	Litlle	No	Much	No	No	Maybe	No	No	No	Very much	No	No	Surelly true	Not true	Not true
Maybe	Litlle	Much	No	No	Litlle	No	No	No	No	No	No	Very much	Litlle	No	Not true	Not true	Not true
Litlle	Maybe	No	No	No	No	No	No	Maybe	No	Litlle	No	No	No	No	Surelly true	moderately true	Not true
No	Maybe	No	No	No	No	No	Much	No	No	No	No	Much	No	Maybe	Surelly true	Surelly true	Not true
No	Maybe	Much	Very much	Much	No	No	No	Maybe	Much	No	No	Maybe	999	Much	Surelly true	Surelly true	moderately true
No	No	No	No	No	No	No	No	No	No	No	No	Maybe	No	No	Surelly true	Surelly true	Not true
No	No	No	No	Maybe	Maybe	No	No	No	No	No	No	Maybe	No	Maybe	Surelly true	Not true	moderately true
No	No	No	No	No	No	No	No	No	No	No	No	Maybe	No	No	Surelly true	Surelly true	Not true
No	No	No	No	Litlle	No	No	No	No	No	No	No	Litlle	No	No	Surelly true	Surelly true	Not true
No	No	No	No	Maybe	No	No	No	No	No	No	No	Litlle	No	No	Surelly true	Surelly true	Not true
Litlle	Maybe	Very much	Litlle	Very much	Very much	No	Litlle	Maybe	No	Very much	Very much	Maybe	Very much	Very much	Surelly true	moderately true	moderately true
Maybe	Litlle	Maybe	Maybe	Very much	Very much	No	Litlle	Maybe	No	Much	Much	Much	Very much	Very much	moderately true	Surelly true	Surelly true
Maybe	Maybe	Much	Litlle	Very much	Very much	No	Litlle	Maybe	No	Very much	Maybe	Much	Very much	Very much	moderately true	Surelly true	Surelly true
Maybe	No	No	No	Maybe	No	No	No	No	No	No	No	No	No	No	Surelly true	moderately true	Not true
No	No	No	No	No	No	No	No	Much	No	Much	No	Much	No	No	Surelly true	Not true	Not true
Much	Maybe	No	Maybe	Much	Much	No	No	No	No	Much	No	Much	No	No	Surelly true	Surelly true	Not true
Much	No	No	No	Much	Much	No	No	Much	No	Much	No	Much	No	Maybe	Surelly true	Surelly true	moderately true
Maybe	No	Maybe	Maybe	Much	Much	No	No	No	No	Much	Maybe	Much	No	Much	Surelly true	Surelly true	moderately true
No	No	No	No	Much	No	No	No	No	No	No	No	Maybe	No	No	moderately true	Surelly true	Not true
No	No	No	No	Much	No	No	No	No	No	No	Much	Much	No	No	Surelly true	Surelly true	Surelly true
Maybe	No	Maybe	No	Maybe	No	No	No	Much	Maybe	No	No	Much	No	No	Surelly true	Surelly true	Not true
Maybe	Much	Maybe	Maybe	Much	Much	No	No	Much	No	Litlle	Maybe	Maybe	No	No	Surelly true	Surelly true	Not true
Maybe	No	Litlle	Litlle	No	Litlle	No	No	Litlle	No	No	No	Litlle	No	No	moderately true	moderately true	Not true
No	No	No	No	Maybe	No	No	No	Litlle	No	No	No	Much	No	No	Surelly true	moderately true	Not true
No	No	No	No	Maybe	No	No	No	No	No	No	No	Maybe	No	No	Surelly true	Not true	Not true
Maybe	Litlle	Litlle	Much	Much	Litlle	No	No	No	No	No	No	Much	No	Maybe	Not true	Surelly true	Surelly true
Maybe	Litlle	No	No	Much	Much	Much	Much	No	No	No	No	Much	No	Maybe	moderately true	Surelly true	Surelly true
No	Litlle	Maybe	Litlle	Litlle	Litlle	Litlle	No	No	No	No	No	Much	No	Maybe	moderately true	Surelly true	Surelly true

Litlle	No	Much	Maybe	No	Much	No	No	Maybe	No	Litlle	Much	Litlle	Maybe	Litlle	Surelly true	Not true	Surelly true
Litlle	No	Much	Maybe	No	No	No	No	Maybe	No	No	No	Litlle	No	No	Surelly true	Surelly true	Not true
Litlle	Much	Much	Maybe	No	Much	No	No	Maybe	No	Litlle	No	Litlle	No	No	Surelly true	Surelly true	Not true
Maybe	No	Litlle	No	Much	Much	No	No	No	No	No	No	Very much	Maybe	Much	Surelly true	moderately true	Surelly true
Litlle	No	Litlle	No	Maybe	Much	No	No	Litlle	No	Litlle	No	Much	No	Maybe	Surelly true	Surelly true	Surelly true
No	No	No	No	No	No	No	No	No	No	No	No	No	No	Litlle	Surelly true	Surelly true	Not true
Maybe	No	Much	Much	Much	No	No	Maybe	No	Very much	No	No	Much	Maybe	Much	Surelly true	Surelly true	Surelly true
No	Much	Much	Much	Much	Much	No	No	Maybe	Maybe	Much	No	Litlle	No	Much	Surelly true	Surelly true	Surelly true
No	No	No	No	Maybe	No	No	No	No	No	No	No	Litlle	No	No	Surelly true	Surelly true	Not true
Much	No	Much	Much	Much	Much	No	No	Maybe	No	Maybe	No	Much	No	Much	Surelly true	Surelly true	Surelly true
No	No	No	No	No	No	No	No	No	No	No	No	Much	No	Litlle	Surelly true	Surelly true	moderately true
Litlle	Litlle	No	Maybe	No	No	No	No	Maybe	Litlle	Very much	No	Much	No	No	Surelly true	moderately true	Not true
No	Maybe	No	No	Maybe	Maybe	No	No	Litlle	No	No	No	Maybe	No	No	Surelly true	moderately true	Not true
Litlle	No	Maybe	No	Maybe	Litlle	No	No	No	No	Litlle	No	Much	No	Maybe	Surelly true	Surelly true	Not true
Litlle	No	Much	No	Maybe	Litlle	No	No	No	No	Litlle	No	Much	No	Maybe	Surelly true	Not true	Not true
No	No	Maybe	No	Maybe	No	No	No	No	No	Litlle	No	Much	No	Maybe	Surelly true	Surelly true	Not true
Maybe	No	Maybe	No	No	No	No	No	Maybe	No	No	No	Very much	No	No	Surelly true	Surelly true	moderately true
No	No	No	No	No	No	No	No	No	No	No	No	Much	No	No	Not true	Surelly true	Not true
No	Maybe	No	Maybe	No	No	No	No	No	No	No	No	Maybe	No	No	moderately true	Surelly true	Not true
No	Very much	No	No	No	No	No	No	No	No	Much	Much	Much	No	Maybe	Surelly true	Surelly true	Surelly true
Maybe	Maybe	Maybe	Litlle	Very much	Very much	No	Maybe	Maybe	No	Much	Maybe	Maybe	Maybe	Maybe	moderately true	moderately true	moderately true
Maybe	No	No	No	Much	Much	No	No	Much	No	Maybe	No	Much	No	Maybe	Surelly true	Surelly true	moderately true
Maybe	No	No	No	Much	Maybe	No	No	No	No	Maybe	No	No	No	Much	Surelly true	moderately true	Surelly true
Maybe	Maybe	Litlle	Litlle	Much	Very much	No	Maybe	Maybe	No	Much	Maybe	Much	Much	Much	moderately true	Surelly true	Surelly true
Very much	Maybe	Maybe	Litlle	Much	Very much	No	Maybe	Maybe	No	Much	Maybe	Very much	Maybe	Maybe	moderately true	moderately true	moderately true
Maybe	Maybe	No	No	No	Maybe	No	No	No	Litlle	Maybe	No	999	Much	Litlle	Surelly true	Surelly true	Surelly true
No	No	No	No	Maybe	No	No	Much	Maybe	No	No	No	Litlle	No	No	Surelly true	moderately true	Not true
No	No	No	No	Litlle	No	No	Much	No	No	No	No	No	No	No	Surelly true	Surelly true	Not true
No	Litlle	Much	Much	Much	Much	No	No	Much	No	Much	No	Much	No	Much	Surelly true	Surelly true	Surelly true
No	No	Litlle	Litlle	Litlle	Much	No	Litlle	Maybe	No	No	Litlle	Litlle	Maybe	Maybe	moderately true	moderately true	moderately true
No	No	Maybe	Maybe	Much	Maybe	No	No	Much	No	Maybe	Maybe	Much	Maybe	Maybe	Surelly true	Surelly true	moderately true
No	No	No	No	Litlle	Litlle	Litlle	Litlle	Litlle	Maybe	Maybe	Litlle	Litlle	Litlle	Litlle	moderately true	Not true	Not true
No	No	No	Litlle	Litlle	Litlle	Maybe	Maybe	Maybe	Litlle	Litlle	Litlle	Litlle	Litlle	No	Surelly true	Not true	Not true
No	No	No	No	No	Litlle	Litlle	Maybe	Maybe	Maybe	Litlle	Litlle	Litlle	Litlle	Litlle	Surelly true	Not true	Not true
No	No	No	No	No	Litlle	Litlle	Maybe	Maybe	Maybe	Litlle	Litlle	Litlle	Litlle	Litlle	Surelly true	Not true	Not true
No	No	No	No	No	Litlle	Litlle	Maybe	Maybe	Maybe	Litlle	Litlle	Litlle	Litlle	Litlle	Surelly true	Not true	Not true
No	No	No	No	No	Litlle	Litlle	Maybe	Maybe	Maybe	Litlle	Litlle	Litlle	Litlle	Litlle	Surelly true	Not true	Not true
Litlle	No	No	No	No	No	No	No	No	No	Litlle	Litlle	Litlle	No	No	Surelly true	Not true	Not true
No	No	No	No	No	Litlle	Litlle	Maybe	Maybe	Maybe	Litlle	Litlle	Litlle	Litlle	Litlle	Surelly true	Not true	Not true
Litlle	No	No	No	No	No	No	No	No	No	Litlle	Litlle	Litlle	No	No	Surelly true	Not true	Not true
No	No	No	Litlle	Litlle	Litlle	Maybe	Maybe	Maybe	Litlle	Litlle	Litlle	Litlle	Litlle	No	Surelly true	Not true	Not true
No	No	No	Litlle	Litlle	Litlle	Maybe	Maybe	Maybe	Litlle	Litlle	Litlle	Litlle	Litlle	No	Surelly true	Not true	Not true
No	No	No	Litlle	Litlle	Litlle	Maybe	Maybe	Maybe	Litlle	Litlle	Litlle	Litlle	Litlle	No	Surelly true	Not true	Not true
No	No	No	Litlle	Litlle	Litlle	Maybe	Maybe	Maybe	Litlle	Litlle	Litlle	Litlle	Litlle	No	Surelly true	Not true	Not true
No	No	No	No	No	No	No	No	No	No	999	No	No	No	No	Surelly true	Not true	Not true
No	No	No	No	No	No	No	No	No	No	No	No	No	No	No	Surelly true	Not true	Not true
Maybe	Maybe	Maybe	Maybe	Maybe	Litlle	Litlle	Much	Much	Maybe	Much	Maybe	Litlle	999	Much	moderately true	moderately true	moderately true
No	No	No	No	Maybe	No	Maybe	No	No	Maybe	Maybe	No	Much	No	No	Surelly true	Surelly true	Not true
Litlle	No	No	No	No	No	No	No	No	No	Litlle	Litlle	Litlle	No	No	Surelly true	Not true	Not true

Litlle	No	No	No	No	No	No	No	No	No	Litlle	Litlle	Litlle	No	No	Surelly true	Not true	Not true
Litlle	No	No	No	No	No	No	No	No	No	Litlle	Litlle	Litlle	No	No	Surelly true	Not true	Not true
No	Litlle	No	No	Litlle	No	Litlle	No	No	No	No	No	Litlle	Maybe	No	Surelly true	Not true	Not true
No	No	Litlle	No	No	No	No	Litlle	No	No	No	No	No	No	No	Surelly true	Not true	Not true
No	No	No	No	No	No	Litlle	Litlle	No	Litlle	No	No	Litlle	No	No	Surelly true	moderately true	Not true
Litlle	No	No	No	No	No	Litlle	Litlle	Litlle	Litlle	Litlle	Litlle	No	No	No	Surelly true	moderately true	moderately true
No	No	No	No	Litlle	Litlle	No	No	No	No	No	Litlle	No	No	No	Surelly true	Not true	Not true
No	No	No	No	No	No	No	No	No	No	No	No	No	No	No	Surelly true	Not true	Not true
No	No	No	No	No	Litlle	Litlle	Maybe	Maybe	Maybe	Litlle	Litlle	Litlle	Litlle	Litlle	Surelly true	Not true	Not true
Litlle	No	No	No	No	No	No	No	No	No	Litlle	Litlle	Litlle	No	No	Surelly true	Not true	Not true
No	No	No	No	No	No	No	No	No	No	No	No	Litlle	Litlle	Litlle	moderately true	moderately true	moderately true
No	No	Maybe	No	Very much	Much	Litlle	No	No	Maybe	No	No	No	No	No	Surelly true	moderately true	Not true
Litlle	Litlle	Maybe	Maybe	Litlle	Litlle	Litlle	No	No	No	No	No	Litlle	No	Litlle	Surelly true	Not true	moderately true
No	No	No	No	No	No	No	No	Maybe	Maybe	Litlle	Maybe	Maybe	Much	Much	9	9	9
No	Litlle	Litlle	Litlle	Litlle	Litlle	No	No	No	No	No	No	No	No	No	moderately true	moderately true	moderately true
No	Litlle	No	No	Litlle	No	Litlle	No	No	No	No	No	Litlle	Maybe	No	Surelly true	Not true	Not true
Maybe	Maybe	Maybe	Maybe	Maybe	Maybe	Litlle	Litlle	Litlle	Litlle	Maybe	Maybe	Litlle	Litlle	Maybe	Surelly true	Not true	Not true
No	No	No	No	Litlle	No	No	No	No	No	No	No	No	No	No	Surelly true	Not true	Not true
No	No	No	No	Litlle	No	No	No	No	No	No	No	No	No	No	Surelly true	Not true	Not true
Litlle	No	No	No	No	No	Litlle	Litlle	Litlle	Litlle	Litlle	Litlle	No	No	No	Surelly true	moderately true	moderately true
Maybe	Litlle	No	Litlle	Maybe	Maybe	Maybe	Much	Very much	Much	Maybe	Maybe	Litlle	No	Litlle	Not true	moderately true	Surelly true
No	No	No	No	Litlle	Litlle	No	No	No	No	No	Litlle	No	No	No	Surelly true	Not true	Not true
No	Maybe	No	No	No	Maybe	Litlle	No	No	No	No	No	No	No	No	Surelly true	moderately true	Surelly true
No	No	No	No	No	No	Litlle	Litlle	No	Litlle	No	No	Litlle	No	No	Surelly true	moderately true	Not true
No	No	No	No	No	No	Litlle	Litlle	Litlle	Litlle	No	No	No	No	Litlle	Surelly true	Not true	Not true
No	No	No	No	No	Litlle	Litlle	Litlle	Litlle	No	No	No	No	Litlle	No	Surelly true	Not true	Not true
No	No	No	Litlle	Litlle	Litlle	Maybe	Maybe	Maybe	Litlle	Litlle	No	No	No	No	Surelly true	Not true	Not true
No	No	No	No	999	Litlle	Litlle	Litlle	No	No	No	No	No	No	No	Surelly true	Not true	Not true
No	No	No	Litlle	Litlle	Litlle	Maybe	Maybe	Maybe	Litlle	Litlle	No	No	No	No	Surelly true	Not true	Not true
No	No	No	No	Litlle	Litlle	Litlle	Litlle	Litlle	Maybe	Maybe	Litlle	Litlle	Litlle	Litlle	moderately true	Not true	Not true
No	No	No	No	No	No	Litlle	Litlle	Litlle	Litlle	No	No	No	No	Litlle	Surelly true	Not true	Not true
No	No	No	Litlle	Litlle	Litlle	Litlle	Litlle	Maybe	Maybe	Litlle	Litlle	Litlle	No	No	moderately true	Not true	Not true
No	No	No	Litlle	Litlle	Litlle	Litlle	Litlle	Maybe	Maybe	Litlle	Litlle	Litlle	No	No	moderately true	Not true	Not true
No	No	No	Litlle	Litlle	Litlle	Litlle	Litlle	Maybe	Maybe	Litlle	Litlle	Litlle	No	No	moderately true	Not true	Not true
No	No	No	No	Litlle	Litlle	Litlle	Litlle	Litlle	Maybe	Maybe	Litlle	Litlle	Litlle	Litlle	moderately true	Not true	Not true
No	No	No	Litlle	Litlle	Litlle	Maybe	Maybe	Maybe	Litlle	Litlle	No	No	No	No	Surelly true	Not true	Not true
No	No	No	No	Litlle	Litlle	Litlle	Litlle	Litlle	Maybe	Maybe	Litlle	Litlle	Litlle	Litlle	moderately true	Not true	Not true
No	No	No	No	Litlle	Litlle	Litlle	Litlle	Litlle	Maybe	Maybe	No	No	No	No	moderately true	Not true	Not true
No	No	No	No	Litlle	Litlle	Litlle	Litlle	Litlle	Maybe	Maybe	Litlle	Litlle	Litlle	Litlle	moderately true	Not true	Not true
No	No	No	No	Litlle	Litlle	Litlle	Litlle	Litlle	Maybe	Maybe	Litlle	Litlle	Litlle	Litlle	moderately true	Not true	Not true
No	No	No	No	Litlle	Litlle	Litlle	Litlle	Litlle	Maybe	Maybe	Litlle	Litlle	Litlle	Litlle	moderately true	moderately true	moderately true
No	No	No	No	Litlle	Litlle	Litlle	Litlle	Litlle	Maybe	Maybe	Litlle	Litlle	Litlle	Litlle	moderately true	Not true	Not true
No	No	No	No	No	Litlle	Litlle	Maybe	Maybe	Maybe	Litlle	Litlle	Litlle	Litlle	Litlle	Surelly true	Not true	Not true
No	No	No	No	No	Litlle	Litlle	Maybe	Maybe	Maybe	Litlle	Litlle	Litlle	Litlle	Litlle	Surelly true	Not true	Not true
No	No	No	Litlle	Litlle	Litlle	Litlle	Litlle	Maybe	Maybe	Litlle	Litlle	Litlle	No	No	moderately true	Not true	Not true
No	No	No	Litlle	Litlle	Litlle	Maybe	Maybe	Maybe	Litlle	Litlle	No	No	No	No	Surelly true	Not true	Not true
No	No	No	Litlle	Litlle	Litlle	Litlle	Litlle	Maybe	Maybe	Litlle	Litlle	Litlle	No	No	moderately true	Not true	Not true
No	No	No	Litlle	Litlle	Litlle	Litlle	Litlle	Maybe	Maybe	Litlle	Litlle	Litlle	No	No	moderately true	Not true	Not true
No	No	No	Litlle	Litlle	Litlle	Maybe	Maybe	Maybe	Litlle	Litlle	No	No	No	No	Surelly true	Not true	Not true

No	No	No	Litlle	Litlle	Litlle	Litlle	Litlle	Maybe	Maybe	Litlle	Litlle	Litlle	No	No	moderately true	Not true	Not true
No	No	No	Litlle	Litlle	Litlle	Litlle	Litlle	Maybe	Maybe	Litlle	Litlle	Litlle	No	No	moderately true	Not true	Not true
No	No	No	Litlle	Litlle	Litlle	Litlle	Litlle	Maybe	Maybe	Litlle	Litlle	Litlle	No	No	moderately true	Not true	Not true
No	No	No	Litlle	Litlle	Litlle	Maybe	Maybe	Maybe	Litlle	Litlle	No	No	No	No	Surelly true	Not true	Not true
No	No	No	Litlle	Litlle	Litlle	Maybe	Maybe	Maybe	Litlle	Litlle	No	No	No	No	Surelly true	Not true	Not true
No	No	No	Litlle	Litlle	Litlle	Maybe	Maybe	Maybe	Litlle	Litlle	No	No	No	No	Surelly true	Not true	Not true
No	No	No	Litlle	Litlle	Litlle	Maybe	Maybe	Maybe	Litlle	Litlle	No	No	No	No	Surelly true	Not true	Not true
999	999	999	999	999	999	999	999	999	999	999	999	999	999	999	9	9	9
999	999	999	999	999	999	999	999	999	999	999	999	999	999	999	9	9	9
No	No	No	No	No	No	Litlle	Litlle	Litlle	Litlle	No	No	No	No	Litlle	Surelly true	Not true	Not true
No	No	No	No	No	No	Litlle	Litlle	Litlle	Litlle	No	No	No	No	Litlle	Surelly true	Not true	Not true
No	No	No	No	No	No	Litlle	Litlle	Litlle	Litlle	No	No	No	No	Litlle	Surelly true	Not true	Not true
No	No	No	No	No	No	Litlle	Litlle	Litlle	Litlle	No	No	No	No	Litlle	Surelly true	Not true	Not true
No	No	No	No	No	No	Litlle	Litlle	Litlle	Litlle	No	No	No	No	Litlle	Surelly true	Not true	Not true
Maybe	Maybe	Maybe	Maybe	Maybe	Maybe	Litlle	Litlle	Litlle	Litlle	Maybe	Maybe	Litlle	Litlle	Maybe	Surelly true	Not true	Not true
Litlle	Litlle	999	Maybe	Maybe	999	Litlle	Litlle	999	No	No	No	999	No	Litlle	Surelly true	Not true	moderately true
No	No	No	No	No	No	No	No	No	No	No	No	No	No	No	Surelly true	Not true	Not true
Maybe	Maybe	No	Litlle	Litlle	Maybe	Maybe	Maybe	Much	Litlle	No	Maybe	Litlle	No	Maybe	Surelly true	moderately true	Not true
Maybe	Maybe	No	Litlle	Litlle	Maybe	Maybe	Maybe	Much	Litlle	999	Maybe	Litlle	No	Maybe	Surelly true	moderately true	Not true
No	No	Maybe	No	Very much	Much	Litlle	No	No	Maybe	No	No	No	No	No	Surelly true	moderately true	Not true
No	Maybe	No	No	No	Maybe	Litlle	No	No	No	No	No	No	No	No	Surelly true	moderately true	Surelly true
Maybe	Litlle	No	Litlle	Maybe	Maybe	Maybe	Much	Very much	Much	Maybe	Maybe	Litlle	No	Litlle	Not true	moderately true	Surelly true
No	No	No	No	No	No	No	No	No	No	No	No	No	No	No	Surelly true	Not true	Not true
Litlle	No	No	No	No	No	No	No	No	No	Litlle	Litlle	Litlle	No	No	Surelly true	Not true	Not true
Litlle	No	No	No	No	No	No	No	No	No	Litlle	Litlle	Litlle	No	No	Surelly true	Not true	Not true
No	No	No	Litlle	Litlle	Litlle	Maybe	Maybe	Maybe	Litlle	Litlle	Litlle	Litlle	Litlle	No	Surelly true	Not true	Not true
No	No	No	Litlle	Litlle	Litlle	Maybe	Maybe	Maybe	Litlle	Litlle	Litlle	Litlle	Litlle	No	Surelly true	Not true	Not true
Litlle	No	No	No	No	No	No	No	No	No	Litlle	Litlle	Litlle	No	No	Surelly true	Not true	Not true
Litlle	No	No	No	No	No	No	No	No	No	Litlle	Litlle	Litlle	No	No	Surelly true	Not true	Not true
No	No	No	No	No	Litlle	Litlle	Maybe	Maybe	Maybe	Litlle	Litlle	Litlle	Litlle	Litlle	Surelly true	Not true	Not true
No	No	No	Litlle	Litlle	Litlle	Maybe	Maybe	Maybe	Litlle	Litlle	Litlle	Litlle	Litlle	No	Surelly true	Not true	Not true
No	No	No	Litlle	Litlle	Litlle	Maybe	Maybe	Maybe	Litlle	Litlle	Litlle	Litlle	Litlle	No	Surelly true	Not true	Not true

b4	b5	b6	b7	b8	b9	b10	b11	b12	b13	b14	b15	b16
Surelly true	Surelly true	moderately true	Surelly true	Not true	Surelly true	Not true	Surelly true	Not true	Not true	Surelly true	Not true	Surelly true
Surelly true	Not true	Not true	Surelly true	Not true	Surelly true	Surelly true	Surelly true	moderately true	Not true	Surelly true	Surelly true	moderately true
Not true	Surelly true	Surelly true	Not true	Surelly true	moderately true	moderately true	moderately true	Surelly true	Surelly true	Surelly true	Surelly true	Surelly true
moderately true	Not true	Not true	Not true	Not true	Surelly true	Surelly true	Surelly true	Surelly true	Not true	Surelly true	moderately true	Surelly true
Not true	Surelly true	moderately true	moderately true	Not true	Surelly true	Surelly true	Surelly true	Surelly true	moderately true	Surelly true	moderately true	Surelly true
moderately true	moderately true	Not true	Surelly true	Not true	Surelly true	Surelly true	Surelly true	Surelly true	moderately true	Surelly true	Not true	Surelly true
Surelly true	Surelly true	Not true	Not true	moderately true	Surelly true	Surelly true	Surelly true	Not true	Surelly true	Surelly true	Not true	Not true
Surelly true	Surelly true	Not true	Surelly true	Surelly true	Surelly true	Surelly true	Surelly true	Not true	moderately true	Surelly true	Not true	Surelly true
moderately true	Surelly true	moderately true	moderately true	Not true	Surelly true	moderately true	Surelly true	Surelly true	moderately true	moderately true	Surelly true	Surelly true
moderately true	Surelly true	moderately true	moderately true	moderately true	moderately true	moderately true	moderately true	moderately true	moderately true	moderately true	moderately true	moderately true
Not true	Surelly true	Not true	Surelly true	Not true	Surelly true	Surelly true	Surelly true	Surelly true	moderately true	Surelly true	moderately true	Surelly true
Surelly true	Surelly true	Not true	moderately true	moderately true	Not true	Surelly true	Surelly true	Surelly true	moderately true	Surelly true	Not true	Surelly true
Surelly true	Not true	Not true	Surelly true	Surelly true	Surelly true	Not true	Surelly true	moderately true	Not true	Surelly true	Not true	Surelly true
Not true	moderately true	moderately true	Surelly true	moderately true	moderately true	Surelly true	Surelly true	Surelly true	moderately true	Surelly true	Surelly true	Surelly true
Surelly true	moderately true	moderately true	moderately true	moderately true	Surelly true	Surelly true	Surelly true	Surelly true	Surelly true	Surelly true	moderately true	moderately true
moderately true	moderately true	Not true	moderately true	Not true	Surelly true	Surelly true	Surelly true	Surelly true	9	Not true	moderately true	Not true
Surelly true	Surelly true	Not true	Surelly true	Not true	Surelly true	Surelly true	moderately true	moderately true	moderately true	Not true	Not true	Surelly true
moderately true	Not true	Not true	moderately true	Not true	Surelly true	Surelly true	Surelly true	Not true	9	moderately true	Surelly true	Not true
Surelly true	Surelly true	Not true	Not true	moderately true	Surelly true	Surelly true	Surelly true	Surelly true	Not true	Surelly true	Surelly true	Surelly true
Surelly true	Surelly true	Not true	moderately true	Not true	moderately true	Surelly true	Surelly true	moderately true	Surelly true	Surelly true	moderately true	moderately true
Surelly true	Surelly true	Not true	Not true	Not true	moderately true	Surelly true	Surelly true	Not true	Not true	moderately true	Surelly true	Surelly true
Not true	Surelly true	moderately true	moderately true	Not true	moderately true	Surelly true	Surelly true	Surelly true	moderately true	Surelly true	Not true	Surelly true
Surelly true	Not true	Not true	moderately true	Not true	Surelly true	Surelly true	Surelly true	moderately true	Not true	Surelly true	moderately true	moderately true
Surelly true	Surelly true	Not true	Surelly true	Not true	Surelly true	Surelly true	Surelly true	Surelly true	Not true	Surelly true	moderately true	Surelly true
Surelly true	Not true	Not true	moderately true	moderately true	Not true	Surelly true	Surelly true	Surelly true	moderately true	Surelly true	moderately true	moderately true
Surelly true	moderately true	Not true	Surelly true	moderately true	Surelly true	Surelly true	Surelly true	Surelly true	Not true	Surelly true	moderately true	Surelly true
Surelly true	Surelly true	moderately true	Surelly true	Surelly true	Surelly true	Surelly true	Surelly true	Not true	Not true	Surelly true	moderately true	Surelly true
Not true	moderately true	Not true	Not true	moderately true	Surelly true	Surelly true	Surelly true	moderately true	moderately true	Surelly true	Not true	Surelly true
moderately true	moderately true	Not true	moderately true	Not true	Surelly true	Surelly true	Surelly true	Surelly true	Not true	Surelly true	Not true	Not true
Surelly true	Surelly true	Not true	Surelly true	Not true	moderately true	Surelly true	Surelly true	Surelly true	Not true	Surelly true	Not true	Surelly true
moderately true	moderately true	moderately true	Surelly true	moderately true	Surelly true	moderately true	moderately true	moderately true	Surelly true	Surelly true	moderately true	moderately true
moderately true	Surelly true	moderately true	Surelly true	Surelly true	moderately true	moderately true	moderately true	moderately true	Surelly true	moderately true	Surelly true	Surelly true
moderately true	Surelly true	moderately true	moderately true	Surelly true	moderately true	Surelly true	moderately true	Surelly true	Surelly true	moderately true	Surelly true	Surelly true
Surelly true	Not true	Not true	Surelly true	moderately true	Surelly true	moderately true	Surelly true	Not true	Not true	Surelly true	Not true	Not true
Surelly true	Surelly true	Not true	moderately true	moderately true	Surelly true	Not true	Surelly true	Surelly true	Surelly true	Surelly true	Surelly true	Surelly true
Surelly true	Surelly true	moderately true	Surelly true	Surelly true	Surelly true	Surelly true	Surelly true	moderately true	Surelly true	Surelly true	Surelly true	Not true
moderately true	Surelly true	moderately true	moderately true	Surelly true	Surelly true	Surelly true	Surelly true	moderately true	Surelly true	Surelly true	Surelly true	Surelly true
Surelly true	Surelly true	Surelly true	Surelly true	Surelly true	Surelly true	Surelly true	Surelly true	Surelly true	Surelly true	Surelly true	moderately true	Surelly true
Surelly true	moderately true	Not true	moderately true	Not true	Surelly true	Surelly true	Surelly true	moderately true	moderately true	Surelly true	Not true	Surelly true
Surelly true	Surelly true	Not true	Surelly true	Not true	Surelly true	Surelly true	Surelly true	Surelly true	Surelly true	Surelly true	Not true	Not true
Surelly true	moderately true	Not true	moderately true	Surelly true	Surelly true	Surelly true	Surelly true	moderately true	moderately true	Surelly true	Surelly true	moderately true
Surelly true	Surelly true	moderately true	Surelly true	Not true	Surelly true	Surelly true	Surelly true	Not true	moderately true	moderately true	Not true	Surelly true
moderately true	Not true	moderately true	moderately true	moderately true	moderately true	9	moderately true	Not true	moderately true	9	moderately true	moderately true
Surelly true	Surelly true	Not true	moderately true	Not true	Surelly true	Not true	Surelly true	Not true	Not true	Surelly true	moderately true	moderately true
Surelly true	Not true	Not true	Surelly true	Not true	Surelly true	Not true	Surelly true	moderately true	Not true	Surelly true	moderately true	Not true
Surelly true	Surelly true	moderately true	moderately true	moderately true	moderately true	Surelly true	Surelly true	moderately true	Surelly true	Surelly true	moderately true	Surelly true
Surelly true	Surelly true	moderately true	moderately true	moderately true	Not true	Surelly true	Surelly true	moderately true	Surelly true	Surelly true	moderately true	moderately true
Surelly true	Surelly true	moderately true	moderately true	moderately true	moderately true	Surelly true	Surelly true	Not true	Surelly true	Surelly true	moderately true	Not true

Surelly true	Surelly true	Not true	moderately true	Surelly true	Surelly true	Not true	Surelly true	Surelly true	Surelly true	Surelly true	Surelly true	Surelly true
moderately true	Surelly true	Not true	moderately true	moderately true	Surelly true	Surelly true	moderately true	Surelly true	Surelly true	Surelly true	Surelly true	Surelly true
moderately true	Surelly true	Not true	moderately true	Surelly true	Surelly true	Surelly true	moderately true	Not true	Surelly true	Surelly true	Surelly true	Surelly true
moderately true	Surelly true	Surelly true	Not true	Surelly true	moderately true	Surelly true	Surelly true	moderately true	Surelly true	Surelly true	Surelly true	Surelly true
moderately true	Surelly true	Not true	moderately true	moderately true	moderately true	Surelly true	Surelly true	Surelly true	Surelly true	Surelly true	Surelly true	Surelly true
Surelly true	Not true	Not true	moderately true	moderately true	Surelly true	Not true	Surelly true	Not true	moderately true	Surelly true	Not true	Not true
Surelly true	Surelly true	Not true	Surelly true	Surelly true	Surelly true	Surelly true	Surelly true	Surelly true	Surelly true	Surelly true	Not true	Not true
Surelly true	moderately true	Not true	Surelly true	Surelly true	Surelly true	Surelly true	moderately true	moderately true	Surelly true	Surelly true	Surelly true	Not true
Surelly true	Surelly true	Not true	moderately true	Not true	Surelly true	moderately true	Surelly true	moderately true	Not true	Surelly true	moderately true	Surelly true
Surelly true	Surelly true	Surelly true	Surelly true	Surelly true	Surelly true	Surelly true	Surelly true	moderately true	Surelly true	Surelly true	Surelly true	Surelly true
Surelly true	Surelly true	Not true	Surelly true	moderately true	Surelly true	Surelly true	Surelly true	Surelly true	Surelly true	Surelly true	Surelly true	Surelly true
Surelly true	Not true	Not true	Surelly true	Not true	Surelly true	Surelly true	Not true	Surelly true	Surelly true	Surelly true	Surelly true	Surelly true
Surelly true	moderately true	Not true	Surelly true	Not true	moderately true	Not true	Surelly true	moderately true	moderately true	Surelly true	moderately true	Not true
Surelly true	moderately true	Not true	Surelly true	moderately true	Surelly true	Surelly true	Surelly true	Not true	Not true	Surelly true	moderately true	Not true
Surelly true	moderately true	Not true	Surelly true	moderately true	Surelly true	Not true	Surelly true	Not true	Not true	Surelly true	Not true	Not true
Surelly true	moderately true	Not true	Surelly true	moderately true	Surelly true	Surelly true	Surelly true	Not true	Not true	Surelly true	moderately true	Not true
Surelly true	Surelly true	Not true	moderately true	Not true	Surelly true	Surelly true	Surelly true	Surelly true	moderately true	Surelly true	moderately true	moderately true
Surelly true	Not true	Not true	moderately true	Not true	Surelly true	Surelly true	Surelly true	Surelly true	Not true	Surelly true	Not true	Not true
Surelly true	moderately true	Not true	moderately true	moderately true	moderately true	Surelly true	Surelly true	Surelly true	Surelly true	Surelly true	moderately true	Surelly true
Not true	Surelly true	Not true	Surelly true	moderately true	Surelly true	Surelly true	Surelly true	Surelly true	Surelly true	Not true	Surelly true	moderately true
Surelly true	Surelly true	moderately true	moderately true	moderately true	moderately true	moderately true	moderately true	moderately true	Surelly true	moderately true	moderately true	moderately true
Surelly true	Surelly true	moderately true	Surelly true	Surelly true	Surelly true	Surelly true	Surelly true	Surelly true	Surelly true	Surelly true	Surelly true	Surelly true
Surelly true	Not true	Not true	Surelly true	moderately true	Surelly true	Not true	Surelly true	Not true	Surelly true	Surelly true	moderately true	Surelly true
Not true	Surelly true	moderately true	moderately true	moderately true	moderately true	Surelly true	moderately true	moderately true	Surelly true	moderately true	moderately true	Surelly true
moderately true	Surelly true	Surelly true	moderately true	moderately true	Not true	Surelly true	Not true	moderately true	Surelly true	moderately true	Surelly true	Surelly true
moderately true	Surelly true	Not true	moderately true	Surelly true	Surelly true	Surelly true	Surelly true	Surelly true	Surelly true	Surelly true	moderately true	Surelly true
Surelly true	Surelly true	Not true	moderately true	Not true	Surelly true	moderately true	Surelly true	Not true	Surelly true	Surelly true	moderately true	Surelly true
Surelly true	moderately true	Not true	moderately true	Not true	moderately true	Surelly true	Surelly true	Not true	Not true	Surelly true	Not true	Not true
Surelly true	Surelly true	moderately true	Not true	moderately true	Surelly true	Surelly true	Surelly true	Surelly true	Surelly true	Surelly true	Surelly true	Surelly true
moderately true	Not true	Not true	moderately true	moderately true	moderately true	Not true	moderately true	Not true	moderately true	moderately true	moderately true	moderately true
Surelly true	Surelly true	Not true	Surelly true	moderately true	Surelly true	Surelly true	Surelly true	Surelly true	Not true	Surelly true	Surelly true	Surelly true
Not true	9	Not true	Not true	Not true	moderately true	Not true	moderately true	Not true	moderately true	moderately true	moderately true	Not true
moderately true	Not true	Not true	moderately true	Not true	moderately true	moderately true	Surelly true	Not true	Not true	moderately true	Not true	Not true
moderately true	Not true	Not true	moderately true	Not true	moderately true	moderately true	Surelly true	Not true	Not true	moderately true	Not true	Not true
moderately true	Not true	Not true	moderately true	Not true	moderately true	moderately true	Surelly true	Not true	Not true	moderately true	Not true	Not true
moderately true	Not true	Not true	moderately true	Not true	moderately true	moderately true	Surelly true	Not true	Not true	moderately true	Not true	Not true
moderately true	Not true	Not true	moderately true	Not true	moderately true	moderately true	Surelly true	Not true	Not true	moderately true	Not true	Not true
moderately true	moderately true	moderately true	Not true	moderately true	moderately true	moderately true	Not true	Not true	moderately true	moderately true	Not true	Not true
moderately true	Not true	Not true	moderately true	Not true	moderately true	moderately true	Surelly true	Not true	Not true	moderately true	Not true	Not true
moderately true	moderately true	Not true	moderately true	Not true	moderately true	moderately true	moderately true	Not true	Not true	moderately true	Not true	Not true
moderately true	Not true	Not true	moderately true	Not true	moderately true	moderately true	Surelly true	Not true	Not true	moderately true	Not true	Not true
moderately true	Not true	Not true	moderately true	Not true	moderately true	moderately true	Surelly true	Not true	Not true	moderately true	Not true	Not true
moderately true	Not true	Not true	moderately true	Not true	moderately true	moderately true	Surelly true	Not true	Not true	moderately true	Not true	Not true
moderately true	Not true	Not true	9	Not true	moderately true	moderately true	Surelly true	Not true	Not true	moderately true	Not true	Not true
moderately true	Not true	Not true	moderately true	Not true	moderately true	Not true	moderately true	Not true	Not true	Not true	Not true	Not true
Surelly true	Surelly true	Not true	moderately true	Not true	Surelly true	moderately true	Surelly true	Not true	Not true	Surelly true	Not true	Not true
Not true	Not true	Not true	moderately true	Not true	moderately true	moderately true	Not true	moderately true	moderately true	moderately true	Surelly true	moderately true
Surelly true	Surelly true	Not true	moderately true	moderately true	Surelly true	Surelly true	Not true	Surelly true	Not true	Surelly true	Not true	Not true
moderately true	moderately true	Not true	moderately true	Not true	moderately true	moderately true	moderately true	Not true	Not true	moderately true	Not true	Not true

moderately true	moderately true	Not true	moderately true	Not true	moderately true	moderately true	moderately true	Not true	Not true	moderately true	Not true	Not true
moderately true	moderately true	Not true	moderately true	Not true	moderately true	moderately true	moderately true	Not true	Not true	moderately true	Not true	Not true
moderately true	Not true	Not true	Surelly true	Not true	moderately true	Not true	Surelly true	moderately true	Not true	Surelly true	Not true	Not true
Surelly true	moderately true	moderately true	Surelly true	moderately true	Surelly true	Not true	Surelly true	moderately true	moderately true	Surelly true	Not true	Not true
moderately true	moderately true	Not true	Surelly true	Not true	Surelly true	moderately true	moderately true	Not true	Not true	Surelly true	Not true	Not true
Surelly true	Not true	moderately true	Surelly true	moderately true	Surelly true	Surelly true	moderately true	Not true	moderately true	moderately true	moderately true	Not true
moderately true	Not true	Not true	moderately true	Not true	Surelly true	Surelly true	Surelly true	moderately true	Not true	Surelly true	moderately true	Not true
Surelly true	moderately true	moderately true	Surelly true	Not true	Surelly true	Not true	Surelly true	moderately true	moderately true	Surelly true	Not true	Not true
moderately true	Not true	Not true	moderately true	Not true	moderately true	moderately true	Surelly true	Not true	Not true	moderately true	Not true	Not true
moderately true	moderately true	Not true	moderately true	Not true	moderately true	moderately true	moderately true	Not true	Not true	moderately true	Not true	Not true
moderately true	Not true	Not true	moderately true	moderately true	moderately true	moderately true	Surelly true	moderately true	moderately true	moderately true	Not true	Not true
Surelly true	moderately true	moderately true	moderately true	moderately true	Not true	Not true	Surelly true	Not true	Surelly true	Surelly true	Not true	Not true
Not true	Surelly true	moderately true	Not true	Not true	Not true	moderately true	moderately true	Surelly true	Surelly true	Surelly true	Surelly true	Surelly true
9	9	9	9	9	9	9	9	9	9	9	9	9
Not true	Not true	Not true	moderately true	Not true	moderately true	Not true	moderately true	moderately true	Not true	moderately true	Not true	Not true
moderately true	Not true	Not true	Surelly true	Not true	moderately true	Not true	Surelly true	moderately true	Not true	Surelly true	Not true	Not true
Surelly true	Not true	Not true	Surelly true	Not true	moderately true	Not true	moderately true	Not true	Not true	Surelly true	Not true	Not true
Not true	Not true	Not true	Not true	moderately true	Not true	Not true	Surelly true	Not true	moderately true	Surelly true	Not true	Not true
Not true	Not true	Not true	moderately true	Not true	Surelly true	Not true	Surelly true	moderately true	Not true	Surelly true	moderately true	Not true
Surelly true	Not true	moderately true	Surelly true	moderately true	Surelly true	Surelly true	moderately true	Not true	moderately true	moderately true	moderately true	Not true
Not true	moderately true	Not true	Surelly true	Surelly true	Surelly true	Not true	moderately true	moderately true	Surelly true	moderately true	Not true	Surelly true
moderately true	Not true	Not true	moderately true	Not true	Surelly true	Surelly true	Surelly true	moderately true	Not true	Surelly true	moderately true	Not true
Surelly true	Surelly true	Surelly true	Surelly true	Surelly true	moderately true	moderately true	Not true	Not true	Surelly true	moderately true	9	Surelly true
moderately true	moderately true	Not true	Surelly true	moderately true	Surelly true	moderately true	moderately true	Not true	Not true	Surelly true	Not true	Not true
moderately true	Not true	Not true	moderately true	Not true	Surelly true	Not true	Surelly true	moderately true	Surelly true	Not true	9	Not true
moderately true	Not true	Not true	moderately true	Not true	Surelly true	Not true	moderately true	Not true	Surelly true	Not true	Not true	moderately true
moderately true	Not true	Not true	moderately true	Not true	Surelly true	moderately true	moderately true	Not true	Not true	moderately true	Not true	Not true
moderately true	moderately true	moderately true	Surelly true	Not true	Surelly true	moderately true	Surelly true	Not true	Not true	moderately true	Not true	Not true
moderately true	Not true	Not true	moderately true	Not true	Surelly true	moderately true	moderately true	Not true	Not true	moderately true	Not true	Not true
Not true	moderately true	Not true	Not true	Not true	Not true	moderately true	Not true	moderately true	moderately true	moderately true	Not true	Not true
moderately true	Not true	Not true	moderately true	Not true	Surelly true	Not true	Surelly true	moderately true	Not true	Surelly true	Not true	Not true
moderately true	Not true	Not true	moderately true	Not true	moderately true	Not true	moderately true	moderately true	Not true	Surelly true	moderately true	Not true
moderately true	Not true	Not true	moderately true	Not true	moderately true	Not true	moderately true	moderately true	Not true	moderately true	moderately true	Not true
moderately true	Not true	Not true	moderately true	Not true	moderately true	Not true	moderately true	moderately true	Not true	Surelly true	moderately true	Not true
Not true	Not true	Not true	Not true	Not true	moderately true	Not true	moderately true	Not true	moderately true	moderately true	moderately true	Not true
moderately true	Not true	Not true	moderately true	Not true	Surelly true	moderately true	moderately true	Not true	Not true	moderately true	Not true	Not true
Not true	moderately true	Not true	Not true	Not true	moderately true	Not true	moderately true	Not true	moderately true	moderately true	moderately true	Not true
Not true	moderately true	Not true	Not true	Not true	moderately true	Not true	moderately true	Not true	moderately true	moderately true	moderately true	Not true
Not true	moderately true	Not true	Not true	Not true	moderately true	Not true	moderately true	Not true	moderately true	moderately true	moderately true	Not true
Not true	moderately true	Not true	Not true	Not true	moderately true	Not true	moderately true	Not true	moderately true	moderately true	moderately true	Not true
Not true	Not true	Not true	Not true	Not true	Not true	Not true	Not true	moderately true	moderately true	moderately true	moderately true	moderately true
Not true	moderately true	Not true	Not true	Not true	moderately true	Not true	moderately true	Not true	moderately true	moderately true	moderately true	Not true
moderately true	Not true	Not true	moderately true	Not true	moderately true	moderately true	Surelly true	Not true	Not true	moderately true	Not true	Not true
moderately true	Not true	Not true	moderately true	Not true	moderately true	moderately true	Surelly true	Not true	Not true	moderately true	Not true	Not true
moderately true	Not true	Not true	moderately true	Not true	moderately true	Not true	moderately true	moderately true	Not true	Surelly true	moderately true	Not true
moderately true	Not true	Not true	moderately true	Not true	Surelly true	moderately true	moderately true	Not true	Not true	moderately true	Not true	Not true
moderately true	Not true	Not true	moderately true	Not true	moderately true	Not true	moderately true	moderately true	Not true	Surelly true	moderately true	Not true
moderately true	Not true	Not true	moderately true	Not true	moderately true	Not true	moderately true	moderately true	Not true	Surelly true	moderately true	Not true
moderately true	Not true	Not true	moderately true	Not true	Surelly true	moderately true	moderately true	Not true	Not true	moderately true	Not true	Not true

moderately true	Not true	Not true	moderately true	Not true	moderately true	Not true	moderately true	moderately true	Not true	Surelly true	moderately true	Not true
moderately true	Not true	Not true	moderately true	Not true	moderately true	Not true	moderately true	moderately true	Not true	Surelly true	moderately true	Not true
moderately true	Not true	Not true	moderately true	Not true	moderately true	Not true	moderately true	moderately true	Not true	Surelly true	moderately true	Not true
moderately true	Not true	Not true	moderately true	Not true	Surelly true	moderately true	moderately true	Not true	Not true	moderately true	Not true	Not true
moderately true	Not true	Not true	moderately true	Not true	Surelly true	moderately true	moderately true	Not true	Not true	moderately true	Not true	Not true
moderately true	Not true	Not true	moderately true	Not true	Surelly true	moderately true	moderately true	Not true	Not true	moderately true	Not true	Not true
moderately true	Not true	Not true	moderately true	Not true	Surelly true	moderately true	moderately true	Not true	Not true	moderately true	Not true	Not true
9	9	9	9	9	9	9	9	9	9	9	9	9
9	9	9	9	9	9	9	9	9	9	9	9	9
moderately true	Not true	Not true	moderately true	Not true	Surelly true	Not true	Surelly true	moderately true	Not true	Surelly true	Not true	Not true
moderately true	Not true	Not true	moderately true	Not true	Surelly true	Not true	9	9	moderately true	Not true	9	Not true
moderately true	9	Not true	Not true	moderately true	Not true	Surelly true	Not true	Surelly true	moderately true	Not true	Surelly true	Not true
moderately true	Not true	Not true	moderately true	Not true	Surelly true	Not true	Surelly true	9	moderately true	Surelly true	Not true	Not true
moderately true	Not true	Not true	moderately true	Not true	Surelly true	Not true	Surelly true	moderately true	Not true	Surelly true	Not true	Not true
Surelly true	Not true	Not true	Surelly true	Not true	moderately true	Not true	moderately true	Not true	Not true	Surelly true	Not true	Not true
Not true	Surelly true	moderately true	Not true	Not true	Not true	moderately true	moderately true	Surelly true	Surelly true	Surelly true	Surelly true	Surelly true
Surelly true	Not true	Not true	Not true	Not true	Not true	moderately true	moderately true	Not true	Not true	Not true	Not true	Not true
Surelly true	Not true	Not true	moderately true	Not true	Surelly true	moderately true	Not true	Not true	Not true	Not true	Not true	Not true
Surelly true	Not true	Not true	moderately true	Not true	Surelly true	moderately true	Not true	Not true	9	Not true	Not true	Not true
Surelly true	moderately true	moderately true	moderately true	moderately true	Not true	Not true	Surelly true	Not true	Surelly true	Surelly true	Not true	Not true
Surelly true	Surelly true	Surelly true	Surelly true	Surelly true	moderately true	moderately true	Not true	Not true	Surelly true	moderately true	9	Surelly true
Not true	moderately true	Not true	Surelly true	Surelly true	Surelly true	Not true	moderately true	moderately true	Surelly true	moderately true	Not true	Surelly true
Surelly true	Not true	Not true	Not true	Not true	Not true	moderately true	moderately true	Not true	Not true	Not true	Not true	Not true
moderately true	moderately true	Not true	moderately true	Not true	moderately true	moderately true	moderately true	Not true	Not true	moderately true	Not true	Not true
moderately true	moderately true	Not true	moderately true	Not true	moderately true	moderately true	moderately true	Not true	Not true	moderately true	Not true	Not true
moderately true	Not true	Not true	9	Not true	moderately true	moderately true	Surelly true	Not true	Not true	9	Not true	Not true
moderately true	Not true	Not true	moderately true	Not true	moderately true	moderately true	Surelly true	Not true	Not true	moderately true	Not true	Not true
moderately true	moderately true	Not true	moderately true	Not true	moderately true	moderately true	moderately true	Not true	Not true	moderately true	Not true	Not true
moderately true	moderately true	Not true	moderately true	Not true	moderately true	moderately true	moderately true	Not true	Not true	moderately true	Not true	Not true
moderately true	Not true	Not true	moderately true	Not true	moderately true	moderately true	Surelly true	Not true	Not true	moderately true	Not true	Not true
9	Not true	Not true	moderately true	Not true	Not true	moderately true	moderately true	Not true	Not true	moderately true	Not true	Not true
9	Not true	Not true	9	Not true	moderately true	moderately true	Surelly true	Not true	Not true	moderately true	Not true	Not true

b17	b18	b19	b20	b21	b22	b23	b24	b25	detentionR	totalPTSD	totalPTSDR	addressR	bb7
Surelly true	Not true	Not true	Surelly true	moderately true	Not true	Surelly true	moderately true	Surelly true	1,00	10,00	score < pc85	refugee camp	Not true
Surelly true	Surelly true	Not true	Surelly true	Not true	Surelly true	Surelly true	Not true	Surelly true	1,00	11,00	score < pc85	refugee camp	Not true
Not true	Surelly true	moderately true	Not true	Not true	Not true	Surelly true	Surelly true	moderately true	1,00	33,00	score > pc85	city	Surelly true
Not true	Surelly true	Not true	Not true	Not true	Surelly true	Surelly true	Surelly true	Surelly true	1,00	17,00	score < pc85	refugee camp	Surelly true
Surelly true	9	Surelly true	moderately true	Not true	moderately true	moderately true	moderately true	moderately true	1,00	20,00	score < pc85	refugee camp	moderately true
Surelly true	Not true	Not true	Surelly true	moderately true	Not true	Surelly true	Surelly true	Surelly true	1,00	11,00	score < pc85	refugee camp	Not true
Surelly true	Not true	Not true	Surelly true	moderately true	Not true	Not true	moderately true	moderately true	1,00	28,00	score < pc85	refugee camp	Surelly true
Surelly true	Not true	Not true	Surelly true	Surelly true	Not true	Not true	moderately true	Surelly true	1,00	19,00	score < pc85	refugee camp	Not true
moderately true	Not true	Not true	Surelly true	Not true	Not true	Surelly true	Not true	Not true	1,00	27,00	score < pc85	city	moderately true
moderately true	moderately true	Not true	moderately true	Not true	moderately true	moderately true	moderately true	Not true	1,00	37,00	score > pc85	village	moderately true
Not true	moderately true	Surelly true	Surelly true	Not true	Not true	Not true	Surelly true	Surelly true	1,00	26,00	score < pc85	refugee camp	Not true
Not true	moderately true	Not true	moderately true	moderately true	Not true	Surelly true	Not true	Surelly true	1,00	26,00	score < pc85	refugee camp	moderately true
Surelly true	moderately true	moderately true	Surelly true	Not true	Not true	Not true	Surelly true	Surelly true	1,00	17,00	score < pc85	refugee camp	Not true
Surelly true	moderately true	Not true	Surelly true	Not true	Not true	moderately true	moderately true	moderately true	1,00	31,00	score > pc85	village	Not true
moderately true	Not true	Not true	Surelly true	moderately true	moderately true	Surelly true	moderately true	Not true	1,00	28,00	score < pc85	village	moderately true
Not true	Not true	Not true	moderately true	Not true	Not true	Surelly true	Not true	Not true	1,00	21,00	score < pc85	city	moderately true
Surelly true	Not true	Not true	Surelly true	Not true	Not true	Not true	Surelly true	Not true	1,00	11,00	score < pc85	refugee camp	Not true
moderately true	Not true	Not true	Surelly true	Not true	Not true	Surelly true	Surelly true	Not true	1,00	17,00	score < pc85	refugee camp	moderately true
Surelly true	Not true	Not true	Surelly true	Not true	Not true	Surelly true	Not true	Not true	1,00	15,00	score < pc85	refugee camp	Surelly true
Surelly true	Not true	Not true	Surelly true	Not true	Not true	moderately true	Surelly true	Not true	1,00	5,00	score < pc85	refugee camp	moderately true
Not true	moderately true	Surelly true	Surelly true	Not true	Not true	Surelly true	moderately true	moderately true	1,00	15,00	score < pc85	refugee camp	Surelly true
Not true	moderately true	Surelly true	moderately true	Not true	Not true	Not true	Surelly true	Surelly true	1,00	22,00	score < pc85	refugee camp	moderately true
Surelly true	moderately true	Not true	moderately true	Not true	Not true	Surelly true	Surelly true	moderately true	1,00	9,00	score < pc85	refugee camp	moderately true
Surelly true	Not true	Not true	moderately true	Not true	Not true	moderately true	Not true	Not true	1,00	12,00	score < pc85	refugee camp	Not true
Surelly true	Not true	Not true	Surelly true	Not true	Not true	Surelly true	moderately true	Surelly true	1,00	#LEEG!	#LEEG!	village	moderately true
Surelly true	Not true	moderately true	Surelly true	Surelly true	Not true	Surelly true	moderately true	Surelly true	1,00	4,00	score < pc85	refugee camp	Not true
Surelly true	Not true	moderately true	Surelly true	Surelly true	Not true	Surelly true	Not true	Surelly true	1,00	21,00	score < pc85	refugee camp	Not true
Not true	Not true	moderately true	moderately true	moderately true	Not true	moderately true	moderately true	Surelly true	1,00	6,00	score < pc85	refugee camp	Surelly true
Surelly true	Not true	Not true	moderately true	Not true	Not true	moderately true	Surelly true	moderately true	1,00	5,00	score < pc85	refugee camp	moderately true
Not true	moderately true	moderately true	Not true	Not true	Not true	Not true	Surelly true	Not true	1,00	4,00	score < pc85	refugee camp	Not true
Surelly true	Not true	Not true	Surelly true	moderately true	Not true	moderately true	Surelly true	Surelly true	1,00	56,00	score > pc85	village	Not true
Not true	Not true	Not true	moderately true	moderately true	Not true	moderately true	Surelly true	moderately true	1,00	54,00	score > pc85	village	Not true
Not true	Not true	moderately true	moderately true	moderately true	Not true	moderately true	Surelly true	moderately true	1,00	57,00	score > pc85	village	moderately true
Surelly true	moderately true	moderately true	Surelly true	moderately true	Not true	Not true	Not true	Surelly true	1,00	9,00	score < pc85	refugee camp	Not true
Not true	Not true	Not true	Surelly true	Not true	Not true	Not true	Surelly true	moderately true	1,00	18,00	score < pc85	refugee camp	moderately true
Surelly true	moderately true	Not true	Surelly true	moderately true	Not true	Surelly true	Not true	Surelly true	1,00	30,00	score > pc85	refugee camp	Not true
Surelly true	moderately true	moderately true	moderately true	Not true	Not true	Not true	Surelly true	moderately true	1,00	26,00	score < pc85	refugee camp	moderately true
Surelly true	moderately true	moderately true	moderately true	moderately true	Not true	Surelly true	Surelly true	moderately true	1,00	39,00	score > pc85	refugee camp	Not true
moderately true	Not true	Not true	moderately true	Not true	Not true	moderately true	moderately true	Not true	1,00	8,00	score < pc85	village	moderately true
Surelly true	Not true	Not true	Surelly true	Not true	Not true	Surelly true	Not true	Surelly true	1,00	19,00	score < pc85	city	Not true
Surelly true	moderately true	moderately true	moderately true	Not true	Not true	moderately true	Not true	moderately true	1,00	20,00	score < pc85	city	moderately true
Surelly true	moderately true	moderately true	Surelly true	Surelly true	Not true	moderately true	Not true	Surelly true	1,00	29,00	score < pc85	city	Not true
moderately true	Not true	moderately true	moderately true	moderately true	Not true	moderately true	moderately true	Not true	1,00	12,00	score < pc85	city	moderately true
Surelly true	Not true	Not true	Surelly true	moderately true	Not true	Surelly true	Not true	moderately true	1,00	10,00	score < pc85	city	moderately true
Surelly true	Not true	moderately true	Surelly true	Surelly true	Not true	Not true	moderately true	moderately true	1,00	5,00	score < pc85	refugee camp	Not true
Not true	moderately true	Not true	Not true	moderately true	Not true	Surelly true	Surelly true	moderately true	1,00	29,00	score < pc85	city	moderately true
Not true	moderately true	Not true	Not true	Not true	Not true	Not true	Surelly true	Not true	1,00	19,00	score < pc85	city	moderately true
Surelly true	Not true	Not true	Not true	Surelly true	Not true	Not true	Not true	moderately true	1,00	18,00	score < pc85	city	moderately true

Surelly true	Not true	Not true	Surelly true	moderately true	Not true	Surelly true	Surelly true	moderately true	1,00	30,00	score > pc85	city	moderately true
Surelly true	moderately true	Not true	Surelly true	Not true	Not true	Surelly true	Surelly true	moderately true	1,00	22,00	score < pc85	city	moderately true
Surelly true	moderately true	Not true	Surelly true	Not true	Not true	Not true	Surelly true	moderately true	1,00	32,00	score > pc85	city	moderately true
Surelly true	moderately true	moderately true	moderately true	moderately true	Not true	Surelly true	Surelly true	moderately true	1,00	32,00	score > pc85	city	Surelly true
moderately true	moderately true	moderately true	Not true	Not true	Not true	Not true	moderately true	Surelly true	1,00	31,00	score > pc85	city	moderately true
Surelly true	Not true	Not true	Surelly true	moderately true	Not true	Not true	Not true	moderately true	1,00	7,00	score < pc85	city	moderately true
Surelly true	Not true	moderately true	Surelly true	Not true	Not true	Surelly true	Surelly true	Surelly true	1,00	37,00	score > pc85	city	Not true
Surelly true	Not true	Not true	Surelly true	Not true	Not true	Surelly true	Surelly true	Surelly true	1,00	41,00	score > pc85	city	Not true
Surelly true	Not true	Not true	moderately true	moderately true	Not true	moderately true	Surelly true	Surelly true	1,00	7,00	score < pc85	village	moderately true
Surelly true	Not true	Not true	Surelly true	moderately true	Not true	moderately true	Surelly true	moderately true	1,00	41,00	score > pc85	village	Not true
Surelly true	Not true	Not true	moderately true	Surelly true	Not true	Surelly true	moderately true	Surelly true	1,00	14,00	score < pc85	village	Not true
Surelly true	Not true	Surelly true	Surelly true	Not true	Not true	Surelly true	Not true	Surelly true	1,00	24,00	score < pc85	village	Not true
Surelly true	Not true	Not true	moderately true	moderately true	Not true	Not true	Not true	moderately true	1,00	11,00	score < pc85	village	Not true
Surelly true	Not true	Not true	Surelly true	Surelly true	Not true	Not true	Surelly true	moderately true	#LEEG!	28,00	score < pc85	village	Not true
Surelly true	Not true	Not true	Surelly true	Surelly true	Not true	Not true	Not true	moderately true	1,00	29,00	score < pc85	village	Not true
Surelly true	Not true	Not true	Surelly true	Surelly true	Not true	Not true	Surelly true	moderately true	1,00	24,00	score < pc85	village	Not true
Surelly true	Not true	moderately true	Surelly true	moderately true	Not true	moderately true	Surelly true	Surelly true	1,00	18,00	score < pc85	village	moderately true
Surelly true	Not true	Not true	Surelly true	Surelly true	Not true	Surelly true	moderately true	Surelly true	1,00	3,00	score < pc85	village	moderately true
Surelly true	Not true	Not true	moderately true	Surelly true	Not true	Not true	Surelly true	moderately true	1,00	9,00	score < pc85	refugee camp	moderately true
Surelly true	moderately true	Not true	Surelly true	Surelly true	Not true	Surelly true	Not true	Not true	1,00	21,00	score < pc85	refugee camp	Not true
moderately true	Not true	Not true	Not true	Not true	Not true	moderately true	Surelly true	Not true	1,00	47,00	score > pc85	village	moderately true
Surelly true	moderately true	moderately true	Surelly true	moderately true	Not true	moderately true	moderately true	Not true	1,00	22,00	score < pc85	refugee camp	Not true
Surelly true	Not true	moderately true	Surelly true	Not true	Not true	Surelly true	Surelly true	Not true	1,00	28,00	score < pc85	village	Not true
moderately true	moderately true	Not true	Not true	Not true	Not true	moderately true	Surelly true	moderately true	1,00	49,00	score > pc85	village	moderately true
Not true	Not true	moderately true	Not true	Not true	Not true	moderately true	Surelly true	Not true	1,00	57,00	score > pc85	village	moderately true
Surelly true	Not true	Not true	moderately true	Not true	Not true	Not true	Surelly true	moderately true	1,00	#LEEG!	#LEEG!	village	moderately true
Surelly true	Not true	Not true	Surelly true	moderately true	Not true	moderately true	Not true	Surelly true	1,00	9,00	score < pc85	village	moderately true
Surelly true	moderately true	Not true	Not true	Not true	Not true	Not true	Not true	Surelly true	1,00	3,00	score < pc85	refugee camp	moderately true
Surelly true	Not true	Not true	Surelly true	moderately true	Not true	Not true	Surelly true	Not true	1,00	39,00	score > pc85	refugee camp	Surelly true
moderately true	Not true	moderately true	moderately true	moderately true	Not true	Not true	moderately true	Not true	1,00	20,00	score < pc85	city	moderately true
Surelly true	moderately true	moderately true	Surelly true	moderately true	Not true	Surelly true	moderately true	Not true	1,00	32,00	score > pc85	refugee camp	Not true
moderately true	Not true	moderately true	moderately true	moderately true	Not true	moderately true	moderately true	moderately true	2,00	12,00	score < pc85	city	Surelly true
moderately true	Not true	Not true	moderately true	moderately true	Not true	moderately true	Not true	moderately true	2,00	12,00	score < pc85	refugee camp	moderately true
moderately true	Not true	Not true	Surelly true	moderately true	Not true	moderately true	Not true	moderately true	2,00	13,00	score < pc85	city	moderately true
moderately true	Not true	Not true	Surelly true	moderately true	Not true	moderately true	Not true	moderately true	2,00	13,00	score < pc85	city	moderately true
moderately true	Not true	Not true	Surelly true	moderately true	Not true	moderately true	Not true	moderately true	2,00	13,00	score < pc85	city	moderately true
moderately true	Not true	Not true	Surelly true	moderately true	Not true	moderately true	Not true	moderately true	2,00	13,00	score < pc85	city	moderately true
moderately true	Not true	moderately true	moderately true	moderately true	Not true	moderately true	Not true	moderately true	2,00	14,00	score < pc85	village	Surelly true
moderately true	Not true	Not true	Surelly true	moderately true	Not true	moderately true	Not true	moderately true	2,00	13,00	score < pc85	city	moderately true
moderately true	Not true	moderately true	moderately true	moderately true	Not true	moderately true	Not true	moderately true	2,00	14,00	score < pc85	village	moderately true
moderately true	Not true	Not true	moderately true	moderately true	Not true	moderately true	Not true	moderately true	2,00	12,00	score < pc85	refugee camp	moderately true
moderately true	Not true	Not true	moderately true	moderately true	Not true	moderately true	Not true	moderately true	2,00	12,00	score < pc85	refugee camp	moderately true
moderately true	Not true	Not true	moderately true	moderately true	Not true	moderately true	Not true	moderately true	2,00	12,00	score < pc85	refugee camp	moderately true
moderately true	Not true	Not true	moderately true	moderately true	Not true	moderately true	Not true	moderately true	2,00	12,00	score < pc85	refugee camp	#LEEG!
moderately true	Not true	Not true	moderately true	Not true	moderately true	Not true	Not true	Not true	2,00	#LEEG!	#LEEG!	village	moderately true
Surelly true	Not true	Not true	Surelly true	Surelly true	Not true	Not true	Not true	Surelly true	2,00	5,00	score < pc85	city	moderately true
moderately true	moderately true	Not true	moderately true	Not true	Surelly true	moderately true	Not true	Not true	2,00	#LEEG!	#LEEG!	village	moderately true
moderately true	Not true	moderately true	Surelly true	Not true	moderately true	Surelly true	Not true	Surelly true	2,00	11,00	score < pc85	refugee camp	moderately true
moderately true	Not true	moderately true	moderately true	moderately true	Not true	moderately true	Not true	moderately true	2,00	14,00	score < pc85	city	moderately true

moderately true	Not true	moderately true	moderately true	moderately true	Not true	moderately true	Not true	moderately true	2,00	14,00	score < pc85	village	moderately true
moderately true	Not true	moderately true	moderately true	moderately true	Not true	moderately true	Not true	moderately true	2,00	14,00	score < pc85	village	moderately true
moderately true	Not true	Not true	Surelly true	moderately true	Not true	moderately true	Not true	Surelly true	2,00	5,00	score < pc85	village	Not true
Surelly true	Not true	Not true	Surelly true	moderately true	Not true	moderately true	Not true	Surelly true	2,00	1,00	score < pc85	village	Not true
Surelly true	Not true	Not true	Surelly true	moderately true	Not true	moderately true	moderately true	moderately true	2,00	1,00	score < pc85	village	Not true
Surelly true	Not true	Not true	Surelly true	Surelly true	Not true	moderately true	moderately true	Surelly true	2,00	5,00	score < pc85	village	Not true
Surelly true	Not true	Not true	Surelly true	moderately true	Not true	moderately true	Not true	Surelly true	2,00	3,00	score < pc85	village	moderately true
Surelly true	Not true	Not true	Surelly true	Not true	moderately true	Not true	moderately true	Surelly true	2,00	0,00	score < pc85	city	Not true
moderately true	Not true	Not true	Surelly true	moderately true	Not true	moderately true	Not true	moderately true	2,00	13,00	score < pc85	city	moderately true
Not true	moderately true	Not true	moderately true	moderately true	Not true	moderately true	Not true	moderately true	2,00	14,00	score < pc85	village	moderately true
moderately true	Not true	Not true	moderately true	moderately true	Not true	moderately true	Not true	moderately true	2,00	7,00	score < pc85	refugee camp	moderately true
Surelly true	Not true	Not true	Surelly true	Surelly true	Not true	Not true	Not true	Surelly true	2,00	13,00	score < pc85	village	moderately true
Not true	Not true	Not true	moderately true	moderately true	moderately true	moderately true	moderately true	moderately true	2,00	10,00	score < pc85	city	Surelly true
9	9	9	9	9	9	9	9	9	2,00	13,00	score < pc85	city	#LEEG!
moderately true	Not true	9	moderately true	moderately true	Not true	Surelly true	Not true	moderately true	2,00	5,00	score < pc85	refugee camp	moderately true
moderately true	Not true	Not true	Surelly true	moderately true	Not true	moderately true	Not true	Surelly true	2,00	5,00	score < pc85	city	Not true
Surelly true	Not true	Not true	Surelly true	Surelly true	Not true	moderately true	Not true	Surelly true	2,00	35,00	score > pc85	city	Not true
moderately true	Not true	Not true	Surelly true	moderately true	Not true	moderately true	Not true	Surelly true	2,00	1,00	score < pc85	village	Surelly true
moderately true	Not true	Not true	Surelly true	moderately true	Not true	moderately true	Not true	Surelly true	2,00	1,00	score < pc85	city	moderately true
Surelly true	Not true	Not true	Surelly true	Surelly true	Not true	moderately true	moderately true	Surelly true	2,00	5,00	score < pc85	refugee camp	Not true
moderately true	moderately true	moderately true	Surelly true	Surelly true	Not true	moderately true	Not true	moderately true	2,00	30,00	score > pc85	village	Not true
Surelly true	Not true	Not true	Surelly true	moderately true	Not true	moderately true	Not true	Surelly true	2,00	3,00	score < pc85	city	moderately true
moderately true	Not true	Surelly true	Surelly true	Surelly true	Surelly true	Surelly true	Surelly true	Surelly true	2,00	5,00	score < pc85	village	Not true
Surelly true	Not true	Not true	Surelly true	moderately true	Not true	moderately true	moderately true	moderately true	2,00	2,00	score < pc85	city	Not true
moderately true	Not true	Not true	moderately true	Surelly true	Not true	moderately true	Not true	moderately true	2,00	8,00	score < pc85	city	moderately true
moderately true	Not true	Not true	moderately true	Surelly true	Not true	moderately true	Not true	moderately true	2,00	9,00	score < pc85	city	moderately true
Surelly true	Not true	Not true	Surelly true	Surelly true	Not true	moderately true	Not true	Surelly true	2,00	11,00	score < pc85	refugee camp	moderately true
moderately true	Not true	Not true	Surelly true	moderately true	Not true	moderately true	Not true	moderately true	2,00	#LEEG!	#LEEG!	village	Not true
Surelly true	Not true	Not true	Surelly true	Surelly true	Not true	moderately true	Not true	Surelly true	2,00	11,00	score < pc85	refugee camp	moderately true
moderately true	Not true	moderately true	moderately true	moderately true	Not true	moderately true	moderately true	moderately true	2,00	12,00	score < pc85	city	Surelly true
moderately true	Not true	Not true	moderately true	Surelly true	Not true	moderately true	Not true	moderately true	2,00	8,00	score < pc85	city	moderately true
moderately true	Not true	Not true	moderately true	moderately true	Not true	Not true	Not true	moderately true	2,00	13,00	score < pc85	village	moderately true
moderately true	Not true	Not true	moderately true	moderately true	Not true	Not true	Not true	moderately true	2,00	13,00	score < pc85	village	moderately true
moderately true	Not true	Not true	moderately true	moderately true	Not true	Not true	Not true	moderately true	2,00	13,00	score < pc85	village	moderately true
moderately true	Not true	moderately true	moderately true	moderately true	Not true	moderately true	moderately true	moderately true	2,00	12,00	score < pc85	city	Surelly true
Surelly true	Not true	Not true	Surelly true	Surelly true	Not true	moderately true	Not true	Surelly true	2,00	11,00	score < pc85	refugee camp	moderately true
moderately true	Not true	moderately true	moderately true	moderately true	Not true	moderately true	moderately true	moderately true	2,00	12,00	score < pc85	city	Surelly true
moderately true	Not true	moderately true	Surelly true	Surelly true	Not true	moderately true	moderately true	moderately true	2,00	8,00	score < pc85	city	Surelly true
moderately true	Not true	moderately true	moderately true	moderately true	Not true	moderately true	moderately true	moderately true	2,00	12,00	score < pc85	city	Surelly true
moderately true	Not true	moderately true	moderately true	moderately true	Not true	moderately true	moderately true	moderately true	2,00	12,00	score < pc85	city	Surelly true
9	9	9	9	9	9	9	9	9	2,00	12,00	score < pc85	city	Surelly true
moderately true	Not true	moderately true	moderately true	moderately true	Not true	moderately true	moderately true	moderately true	2,00	12,00	score < pc85	city	Surelly true
moderately true	Not true	Not true	Surelly true	moderately true	Not true	moderately true	Not true	moderately true	2,00	13,00	score < pc85	city	moderately true
moderately true	Not true	Not true	Surelly true	moderately true	Not true	moderately true	Not true	moderately true	2,00	13,00	score < pc85	city	moderately true
moderately true	Not true	Not true	moderately true	moderately true	Not true	Not true	Not true	moderately true	2,00	13,00	score < pc85	village	moderately true
Surelly true	Not true	Not true	Surelly true	Surelly true	Not true	moderately true	Not true	Surelly true	2,00	11,00	score < pc85	refugee camp	moderately true
moderately true	Not true	Not true	moderately true	moderately true	Not true	Not true	Not true	moderately true	2,00	13,00	score < pc85	village	moderately true
moderately true	Not true	Not true	moderately true	moderately true	Not true	Not true	Not true	moderately true	2,00	13,00	score < pc85	village	moderately true
Surelly true	Not true	Not true	Surelly true	Surelly true	Not true	moderately true	Not true	Surelly true	2,00	11,00	score < pc85	refugee camp	moderately true

moderately true	Not true	Not true	moderately true	moderately true	Not true	Not true	Not true	moderately true	2,00	13,00	score < pc85	village	moderately true
moderately true	Not true	Not true	moderately true	moderately true	Not true	Not true	Not true	moderately true	2,00	13,00	score < pc85	village	moderately true
moderately true	Not true	Not true	moderately true	moderately true	Not true	Not true	Not true	moderately true	2,00	13,00	score < pc85	village	moderately true
Surelly true	Not true	Not true	Surelly true	Surelly true	Not true	moderately true	Not true	Surelly true	2,00	11,00	score < pc85	refugee camp	moderately true
Surelly true	Not true	Not true	Surelly true	Surelly true	Not true	moderately true	Not true	Surelly true	2,00	11,00	score < pc85	refugee camp	moderately true
Surelly true	Not true	Not true	Surelly true	Surelly true	Not true	moderately true	Not true	Surelly true	2,00	11,00	score < pc85	refugee camp	moderately true
Surelly true	Not true	Not true	Surelly true	Surelly true	Not true	moderately true	Not true	Surelly true	2,00	11,00	score < pc85	refugee camp	moderately true
9	9	9	9	9	9	9	9	9	2,00	#LEEG!	#LEEG!	city	#LEEG!
9	9	9	9	9	9	9	9	9	2,00	#LEEG!	#LEEG!	city	#LEEG!
moderately true	Not true	Not true	moderately true	Surelly true	Not true	moderately true	Not true	moderately true	2,00	8,00	score < pc85	city	moderately true
moderately true	Not true	Not true	moderately true	Surelly true	Not true	moderately true	Not true	moderately true	2,00	8,00	score < pc85	city	moderately true
moderately true	Not true	Not true	moderately true	Surelly true	Not true	moderately true	Not true	moderately true	2,00	8,00	score < pc85	city	Surelly true
moderately true	Not true	Not true	moderately true	Surelly true	Not true	9	Not true	moderately true	2,00	8,00	score < pc85	city	moderately true
moderately true	Not true	Not true	moderately true	Surelly true	Not true	moderately true	Not true	moderately true	2,00	8,00	score < pc85	city	moderately true
Surelly true	Not true	Not true	Surelly true	Surelly true	Not true	moderately true	Not true	Surelly true	2,00	35,00	score > pc85	village	Not true
Not true	Not true	Not true	9	moderately true	moderately true	moderately true	moderately true	moderately true	2,00	#LEEG!	#LEEG!	refugee camp	Surelly true
Not true	Not true	Not true	Not true	moderately true	Not true	moderately true	Not true	Surelly true	2,00	0,00	score < pc85	refugee camp	Surelly true
Not true	Not true	Not true	Not true	Not true	Not true	Not true	Not true	Not true	2,00	20,00	score < pc85	city	moderately true
Not true	Not true	Not true	Not true	Not true	Not true	Not true	Not true	Not true	2,00	#LEEG!	#LEEG!	refugee camp	moderately true
Surelly true	Not true	Not true	Surelly true	Surelly true	Not true	Not true	Not true	Surelly true	2,00	13,00	score < pc85	refugee camp	moderately true
moderately true	Not true	Surelly true	Surelly true	Surelly true	Surelly true	Surelly true	Surelly true	Surelly true	2,00	5,00	score < pc85	refugee camp	Not true
moderately true	moderately true	moderately true	Surelly true	Surelly true	Not true	moderately true	Surelly true	moderately true	2,00	32,00	score > pc85	refugee camp	Not true
Not true	Not true	Not true	Not true	moderately true	Not true	moderately true	Not true	Surelly true	2,00	0,00	score < pc85	village	Surelly true
moderately true	9	moderately true	moderately true	moderately true	Not true	moderately true	Not true	moderately true	2,00	14,00	score < pc85	village	moderately true
moderately true	Not true	moderately true	moderately true	moderately true	Not true	moderately true	Not true	moderately true	2,00	14,00	score < pc85	village	moderately true
moderately true	Not true	Not true	moderately true	moderately true	Not true	moderately true	Not true	moderately true	2,00	12,00	score < pc85	refugee camp	#LEEG!
moderately true	Not true	Not true	moderately true	moderately true	Not true	moderately true	Not true	moderately true	2,00	12,00	score < pc85	refugee camp	moderately true
moderately true	Not true	moderately true	moderately true	moderately true	Not true	moderately true	Not true	moderately true	2,00	14,00	score < pc85	village	moderately true
moderately true	Not true	moderately true	moderately true	moderately true	Not true	moderately true	Not true	moderately true	2,00	14,00	score < pc85	village	moderately true
moderately true	Not true	Not true	Surelly true	moderately true	Not true	moderately true	Not true	moderately true	2,00	13,00	score < pc85	city	moderately true
moderately true	Not true	Not true	moderately true	moderately true	Not true	moderately true	Not true	moderately true	2,00	12,00	score < pc85	refugee camp	moderately true
moderately true	Not true	Not true	moderately true	moderately true	Not true	moderately true	Not true	moderately true	2,00	12,00	score < pc85	refugee camp	#LEEG!

bb11	bb14	bb21	bb25	filter_$	viewdetentionR	totalsdq	emotionalsdq	hyperactivitysdq	prosocialsdq	totalsdqR	totalPTSDadjusted	totalsdqadjusted	hyperactivitysdqR
Not true	Not true	moderately true	Not true	Not Selected	no	10,00	4,00	1,00	10,00	0,00	220,00	200,00	0,00
Not true	Not true	Surelly true	Not true	Not Selected	no	12,00	1,00	6,00	9,00	0,00	242,00	240,00	0,00
moderately true	Not true	Surelly true	moderately true	Not Selected	no	28,00	10,00	7,00	3,00	1,00	726,00	560,00	0,00
Not true	Not true	Surelly true	Not true	Not Selected	no	17,00	4,00	7,00	5,00	0,00	374,00	340,00	0,00
Not true	Not true	Surelly true	moderately true	Not Selected	no	21,33	4,00	8,00	6,00	1,00	440,00	426,67	1,00
Not true	Not true	moderately true	Not true	Not Selected	no	17,00	7,00	5,00	9,00	0,00	242,00	340,00	0,00
Not true	Not true	moderately true	moderately true	Not Selected	yes	12,00	4,00	6,00	10,00	0,00	616,00	240,00	0,00
Not true	Not true	Not true	Not true	Not Selected	yes	12,00	6,00	4,00	10,00	0,00	418,00	240,00	0,00
Not true	moderately true	Surelly true	Surelly true	Not Selected	no	17,00	3,00	7,00	7,00	0,00	594,00	340,00	0,00
moderately true	moderately true	Surelly true	Surelly true	Not Selected	no	18,00	5,00	7,00	5,00	0,00	814,00	360,00	0,00
Not true	Not true	Surelly true	Not true	Not Selected	no	19,00	5,00	7,00	4,00	0,00	572,00	380,00	0,00
Not true	Not true	moderately true	Not true	Not Selected	no	16,00	4,00	5,00	3,00	0,00	572,00	320,00	0,00
Not true	Not true	Surelly true	Not true	Not Selected	no	13,00	6,00	4,00	10,00	0,00	374,00	260,00	0,00
Not true	Not true	Surelly true	moderately true	Not Selected	yes	21,00	6,00	9,00	7,00	1,00	682,00	420,00	1,00
Not true	Not true	moderately true	Surelly true	Not Selected	no	17,00	5,00	6,00	9,00	0,00	616,00	340,00	0,00
Not true	Surelly true	Surelly true	Surelly true	Not Selected	no	14,93	0,00	9,00	5,00	0,00	462,00	298,67	1,00
moderately true	Surelly true	Surelly true	Surelly true	Not Selected	no	16,00	5,00	8,00	10,00	0,00	242,00	320,00	1,00
Not true	moderately true	Surelly true	Surelly true	Not Selected	no	12,80	2,50	8,00	8,00	0,00	374,00	256,00	1,00
Not true	Not true	Surelly true	Surelly true	Not Selected	no	20,00	4,00	10,00	10,00	1,00	330,00	400,00	1,00
Not true	Not true	Surelly true	Surelly true	Not Selected	yes	18,00	5,00	9,00	9,00	0,00	110,00	360,00	1,00
Not true	moderately true	Surelly true	moderately true	Not Selected	yes	17,00	3,00	7,00	7,00	0,00	330,00	340,00	0,00
Not true	Not true	Surelly true	Not true	Not Selected	yes	17,00	5,00	4,00	2,00	0,00	484,00	340,00	0,00
Not true	Not true	Surelly true	moderately true	Not Selected	yes	14,00	3,00	7,00	9,00	0,00	198,00	280,00	0,00
Not true	Not true	Surelly true	Surelly true	Not Selected	no	16,00	2,00	9,00	9,00	0,00	264,00	320,00	1,00
Not true	Not true	Surelly true	Not true	Not Selected	no	16,00	5,00	7,00	8,00	0,00	#LEEG!	320,00	0,00
Not true	Not true	Not true	Not true	Not Selected	no	15,00	4,00	5,00	10,00	0,00	88,00	300,00	0,00
Not true	Not true	Not true	Not true	Not Selected	no	14,00	5,00	3,00	10,00	0,00	462,00	280,00	0,00
Not true	Not true	moderately true	Not true	Not Selected	no	14,00	5,00	5,00	5,00	0,00	132,00	280,00	0,00
Not true	Not true	Surelly true	moderately true	Not Selected	no	13,00	2,00	7,00	8,00	0,00	110,00	260,00	0,00
Not true	Not true	Surelly true	Surelly true	Not Selected	yes	18,00	4,00	8,00	5,00	0,00	88,00	360,00	1,00
moderately true	Not true	moderately true	Not true	Not Selected	yes	15,00	7,00	4,00	9,00	0,00	1232,00	300,00	0,00
moderately true	moderately true	moderately true	moderately true	Not Selected	yes	22,00	10,00	7,00	4,00	1,00	1188,00	440,00	0,00
moderately true	moderately true	moderately true	moderately true	Not Selected	yes	25,00	10,00	8,00	4,00	1,00	1254,00	500,00	1,00
Not true	Not true	moderately true	Not true	Not Selected	no	6,00	1,00	3,00	10,00	0,00	198,00	120,00	0,00
Not true	Not true	Surelly true	moderately true	Not Selected	no	16,00	7,00	5,00	8,00	0,00	396,00	320,00	0,00
Not true	Not true	moderately true	Not true	Not Selected	no	18,00	4,00	7,00	10,00	0,00	660,00	360,00	0,00
Not true	Not true	Surelly true	moderately true	Not Selected	no	24,00	9,00	9,00	8,00	1,00	572,00	480,00	1,00
Not true	Not true	moderately true	moderately true	Not Selected	no	26,00	9,00	7,00	9,00	1,00	858,00	520,00	0,00
Not true	Not true	Surelly true	Surelly true	Not Selected	no	15,00	4,00	8,00	7,00	0,00	176,00	300,00	1,00
Not true	Not true	Surelly true	Not true	Not Selected	no	16,00	4,00	6,00	10,00	0,00	418,00	320,00	0,00
Not true	Not true	Surelly true	moderately true	Not Selected	no	18,00	4,00	9,00	9,00	0,00	440,00	360,00	1,00
Not true	moderately true	Not true	Not true	Not Selected	yes	13,00	3,00	4,00	10,00	0,00	638,00	260,00	0,00
moderately true	#LEEG!	moderately true	Surelly true	Not Selected	no	12,80	4,00	6,25	5,00	0,00	264,00	256,00	0,00
Not true	Not true	moderately true	moderately true	Not Selected	no	11,00	1,00	4,00	10,00	0,00	220,00	220,00	0,00
Not true	Not true	Not true	moderately true	Not Selected	no	5,00	1,00	2,00	10,00	0,00	110,00	100,00	0,00
Not true	Not true	moderately true	moderately true	Not Selected	yes	23,00	9,00	7,00	3,00	1,00	638,00	460,00	0,00
Not true	Not true	Surelly true	Surelly true	Not Selected	yes	22,00	8,00	9,00	3,00	1,00	418,00	440,00	1,00
Not true	Not true	Not true	moderately true	Not Selected	no	14,00	5,00	6,00	6,00	0,00	396,00	280,00	0,00

Not true	Not true	moderately true	moderately true	Not Selected	yes		20,00	10,00	4,00	10,00	1,00	660,00	400,00	0,00
moderately true	Not true	Surelly true	moderately true	Not Selected	yes		23,00	7,00	9,00	9,00	1,00	484,00	460,00	1,00
moderately true	Not true	Surelly true	moderately true	Not Selected	yes		20,00	8,00	9,00	9,00	1,00	704,00	400,00	1,00
Not true	Not true	moderately true	moderately true	Not Selected	no		26,00	10,00	7,00	7,00	1,00	704,00	520,00	0,00
Not true	Not true	Surelly true	Not true	Not Selected	no		22,00	8,00	8,00	5,00	1,00	682,00	440,00	1,00
Not true	Not true	moderately true	moderately true	Not Selected	no		6,00	2,00	4,00	10,00	0,00	154,00	120,00	0,00
Not true	Not true	Surelly true	Not true	Not Selected	yes		21,00	8,00	6,00	10,00	1,00	814,00	420,00	0,00
moderately true	Not true	Surelly true	Not true	Not Selected	yes		20,00	8,00	8,00	10,00	1,00	902,00	400,00	1,00
Not true	Not true	moderately true	Not true	Not Selected	no		13,00	4,00	5,00	9,00	0,00	154,00	260,00	0,00
Not true	Not true	moderately true	moderately true	Not Selected	yes		24,00	10,00	8,00	10,00	1,00	902,00	480,00	1,00
Not true	Not true	Not true	Not true	Not Selected	no		19,00	7,00	6,00	9,00	0,00	308,00	380,00	0,00
Surelly true	Not true	Surelly true	Not true	Not Selected	no		17,00	4,00	7,00	10,00	0,00	528,00	340,00	0,00
Not true	Not true	moderately true	moderately true	Not Selected	yes		7,00	1,00	4,00	8,00	0,00	242,00	140,00	0,00
Not true	Not true	Not true	moderately true	Not Selected	#LEEG!		10,00	3,00	6,00	10,00	0,00	616,00	200,00	0,00
Not true	Not true	Not true	moderately true	Not Selected	yes		3,00	1,00	1,00	10,00	0,00	638,00	60,00	0,00
Not true	Not true	Not true	moderately true	Not Selected	no		10,00	3,00	6,00	10,00	0,00	528,00	200,00	0,00
Not true	Not true	moderately true	Not true	Not Selected	no		17,00	5,00	6,00	10,00	0,00	396,00	340,00	0,00
Not true	Not true	Not true	Not true	Not Selected	no		9,00	1,00	4,00	8,00	0,00	66,00	180,00	0,00
Not true	Not true	Not true	moderately true	Not Selected	no		16,00	7,00	6,00	7,00	0,00	198,00	320,00	0,00
Not true	Surelly true	Not true	Surelly true	Not Selected	no		21,00	6,00	8,00	8,00	1,00	462,00	420,00	1,00
moderately true	moderately true	Surelly true	Surelly true	Not Selected	yes		19,00	7,00	7,00	5,00	0,00	1034,00	380,00	0,00
Not true	Not true	moderately true	Surelly true	Not Selected	no		25,00	8,00	9,00	10,00	1,00	484,00	500,00	1,00
Not true	Not true	Surelly true	Surelly true	Not Selected	yes		18,00	9,00	6,00	10,00	0,00	616,00	360,00	0,00
moderately true	moderately true	Surelly true	moderately true	Not Selected	yes		23,00	9,00	8,00	3,00	1,00	1078,00	460,00	1,00
Surelly true	moderately true	Surelly true	Surelly true	Not Selected	yes		24,00	8,00	9,00	2,00	1,00	1254,00	480,00	1,00
Not true	Not true	Surelly true	moderately true	Not Selected	yes		22,00	10,00	8,00	8,00	1,00	#LEEG!	440,00	1,00
Not true	Not true	moderately true	Not true	Not Selected	no		11,00	4,00	4,00	10,00	0,00	198,00	220,00	0,00
Not true	Not true	Surelly true	Not true	Not Selected	no		8,00	0,00	6,00	7,00	0,00	66,00	160,00	0,00
Not true	Not true	moderately true	Surelly true	Not Selected	no		23,00	9,00	9,00	10,00	1,00	858,00	460,00	1,00
moderately true	moderately true	moderately true	Surelly true	Not Selected	yes		11,00	5,00	5,00	5,00	0,00	440,00	220,00	0,00
Not true	Not true	moderately true	Surelly true	Not Selected	yes		22,00	5,00	9,00	10,00	1,00	704,00	440,00	1,00
moderately true	moderately true	moderately true	moderately true	Selected		999,00	7,47	2,00	3,00	4,00	0,00	264,00	149,33	0,00
Not true	moderately true	moderately true	moderately true	Selected		999,00	4,00	0,00	3,00	6,00	0,00	264,00	80,00	0,00
Not true	moderately true	moderately true	moderately true	Selected		999,00	4,00	0,00	3,00	7,00	0,00	286,00	80,00	0,00
Not true	moderately true	moderately true	moderately true	Selected		999,00	4,00	0,00	3,00	7,00	0,00	286,00	80,00	0,00
Not true	moderately true	moderately true	moderately true	Selected		999,00	4,00	0,00	3,00	7,00	0,00	286,00	80,00	0,00
Not true	moderately true	moderately true	moderately true	Selected		999,00	4,00	0,00	3,00	7,00	0,00	286,00	80,00	0,00
Surelly true	moderately true	moderately true	moderately true	Selected		999,00	9,00	2,00	3,00	6,00	0,00	308,00	180,00	0,00
Not true	moderately true	moderately true	moderately true	Selected		999,00	4,00	0,00	3,00	7,00	0,00	286,00	80,00	0,00
moderately true	moderately true	moderately true	moderately true	Selected		999,00	6,00	0,00	3,00	6,00	0,00	308,00	120,00	0,00
Not true	moderately true	moderately true	moderately true	Selected		999,00	4,00	0,00	3,00	6,00	0,00	264,00	80,00	0,00
Not true	moderately true	moderately true	moderately true	Selected		999,00	4,00	0,00	3,00	6,00	0,00	264,00	80,00	0,00
Not true	moderately true	moderately true	moderately true	Selected		999,00	4,00	0,00	3,00	6,00	0,00	264,00	80,00	0,00
Not true	moderately true	moderately true	moderately true	Selected		999,00	4,00	0,00	3,00	6,00	0,00	264,00	80,00	0,00
moderately true	Surelly true	Surelly true	Surelly true	Selected		999,00	4,00	0,00	4,00	6,00	0,00	#LEEG!	80,00	0,00
Not true	Not true	Not true	Not true	Selected		999,00	3,00	0,00	1,00	10,00	0,00	110,00	60,00	0,00
Surelly true	moderately true	Surelly true	Surelly true	Selected		999,00	14,00	3,00	8,00	4,00	0,00	#LEEG!	280,00	1,00
Surelly true	Not true	Surelly true	Not true	Selected		999,00	14,00	1,00	6,00	9,00	0,00	242,00	280,00	0,00
moderately true	moderately true	moderately true	moderately true	Selected		999,00	6,00	0,00	3,00	6,00	0,00	308,00	120,00	0,00

moderately true	moderately true	moderately true	moderately true	Selected	999,00	6,00	0,00	3,00	6,00	0,00	308,00	120,00	0,00
moderately true	moderately true	moderately true	moderately true	Selected	999,00	6,00	0,00	3,00	6,00	0,00	308,00	120,00	0,00
Not true	Not true	moderately true	Not true	Selected	999,00	3,00	0,00	1,00	7,00	0,00	110,00	60,00	0,00
Not true	Not true	moderately true	Not true	Selected	999,00	7,00	2,00	1,00	10,00	0,00	22,00	140,00	0,00
moderately true	Not true	moderately true	moderately true	Selected	999,00	7,00	1,00	4,00	9,00	0,00	22,00	140,00	0,00
moderately true	moderately true	Not true	Not true	Selected	999,00	10,00	4,00	4,00	10,00	0,00	110,00	200,00	0,00
Not true	Not true	moderately true	Not true	Selected	999,00	6,00	0,00	4,00	9,00	0,00	66,00	120,00	0,00
Not true	Not true	Surelly true	Not true	Selected	999,00	7,00	2,00	2,00	10,00	0,00	0,00	140,00	0,00
Not true	moderately true	moderately true	moderately true	Selected	999,00	4,00	0,00	3,00	7,00	0,00	286,00	80,00	0,00
moderately true	moderately true	moderately true	moderately true	Selected	999,00	6,00	0,00	3,00	5,00	0,00	308,00	120,00	0,00
Not true	moderately true	moderately true	moderately true	Selected	999,00	9,00	3,00	4,00	5,00	0,00	154,00	180,00	0,00
Not true	Not true	Not true	Not true	Selected	999,00	6,00	3,00	1,00	8,00	0,00	286,00	120,00	0,00
moderately true	Not true	moderately true	moderately true	Selected	999,00	17,00	6,00	5,00	3,00	0,00	220,00	340,00	0,00
#LEEG!	#LEEG!	#LEEG!	#LEEG!	Selected	999,00	#LEEG!	#LEEG!	#LEEG!	#LEEG!	#LEEG!	286,00	#LEEG!	#LEEG!
moderately true	moderately true	moderately true	moderately true	Selected	999,00	7,47	1,00	3,00	4,00	0,00	110,00	149,33	0,00
Not true	Not true	moderately true	Not true	Selected	999,00	3,00	0,00	1,00	7,00	0,00	110,00	60,00	0,00
moderately true	Not true	Not true	Not true	Selected	999,00	1,00	0,00	0,00	9,00	0,00	770,00	20,00	0,00
Not true	Not true	moderately true	Not true	Selected	999,00	4,00	2,00	1,00	5,00	0,00	22,00	80,00	0,00
Not true	Not true	moderately true	Not true	Selected	999,00	4,00	0,00	2,00	7,00	0,00	22,00	80,00	0,00
moderately true	moderately true	Not true	Not true	Selected	999,00	10,00	4,00	4,00	10,00	0,00	110,00	200,00	0,00
moderately true	moderately true	Not true	moderately true	Selected	999,00	15,00	8,00	2,00	5,00	0,00	660,00	300,00	0,00
Not true	Not true	moderately true	Not true	Selected	999,00	6,00	0,00	4,00	9,00	0,00	66,00	120,00	0,00
Surelly true	moderately true	Not true	Not true	Selected	999,00	21,33	10,00	2,50	8,00	1,00	110,00	426,67	0,00
moderately true	Not true	moderately true	moderately true	Selected	999,00	8,00	2,00	4,00	9,00	0,00	44,00	160,00	0,00
Not true	Surelly true	Not true	moderately true	Selected	999,00	5,33	2,00	1,25	7,00	0,00	176,00	106,67	0,00
moderately true	Surelly true	Not true	moderately true	Selected	999,00	5,00	3,00	1,00	7,00	0,00	198,00	100,00	0,00
moderately true	moderately true	Not true	Not true	Selected	999,00	2,00	0,00	1,00	9,00	0,00	242,00	40,00	0,00
Not true	moderately true	moderately true	moderately true	Selected	999,00	6,00	0,00	3,00	8,00	0,00	#LEEG!	120,00	0,00
moderately true	moderately true	Not true	Not true	Selected	999,00	2,00	0,00	1,00	9,00	0,00	242,00	40,00	0,00
Surelly true	moderately true	moderately true	moderately true	Selected	999,00	9,00	2,00	3,00	3,00	0,00	264,00	180,00	0,00
Not true	Not true	Not true	moderately true	Selected	999,00	3,00	0,00	1,00	7,00	0,00	176,00	60,00	0,00
moderately true	Not true	moderately true	moderately true	Selected	999,00	4,00	0,00	3,00	5,00	0,00	286,00	80,00	0,00
moderately true	moderately true	moderately true	moderately true	Selected	999,00	4,00	0,00	3,00	5,00	0,00	286,00	80,00	0,00
moderately true	Not true	moderately true	moderately true	Selected	999,00	4,00	0,00	3,00	5,00	0,00	286,00	80,00	0,00
moderately true	moderately true	moderately true	moderately true	Selected	999,00	7,00	2,00	3,00	4,00	0,00	264,00	140,00	0,00
moderately true	moderately true	Not true	Not true	Selected	999,00	2,00	0,00	1,00	9,00	0,00	242,00	40,00	0,00
moderately true	moderately true	moderately true	moderately true	Selected	999,00	8,00	2,00	3,00	4,00	0,00	264,00	160,00	0,00
moderately true	moderately true	Not true	moderately true	Selected	999,00	7,00	2,00	2,00	5,00	0,00	176,00	140,00	0,00
moderately true	moderately true	moderately true	moderately true	Selected	999,00	8,00	2,00	3,00	4,00	0,00	264,00	160,00	0,00
moderately true	moderately true	moderately true	moderately true	Selected	999,00	8,00	2,00	3,00	4,00	0,00	264,00	160,00	0,00
Surelly true	moderately true	#LEEG!	#LEEG!	Selected	999,00	9,60	3,75	3,33	1,67	0,00	264,00	192,00	0,00
moderately true	moderately true	moderately true	moderately true	Selected	999,00	8,00	2,00	3,00	4,00	0,00	264,00	160,00	0,00
Not true	moderately true	moderately true	moderately true	Selected	999,00	4,00	0,00	3,00	7,00	0,00	286,00	80,00	0,00
Not true	moderately true	moderately true	moderately true	Selected	999,00	4,00	0,00	3,00	7,00	0,00	286,00	80,00	0,00
moderately true	Not true	moderately true	moderately true	Selected	999,00	4,00	0,00	3,00	5,00	0,00	286,00	80,00	0,00
moderately true	moderately true	Not true	Not true	Selected	999,00	2,00	0,00	1,00	9,00	0,00	242,00	40,00	0,00
moderately true	Not true	moderately true	moderately true	Selected	999,00	4,00	0,00	3,00	5,00	0,00	286,00	80,00	0,00
moderately true	Not true	moderately true	moderately true	Selected	999,00	4,00	0,00	3,00	5,00	0,00	286,00	80,00	0,00
moderately true	moderately true	Not true	Not true	Selected	999,00	2,00	0,00	1,00	9,00	0,00	242,00	40,00	0,00

moderately true	Not true	moderately true	moderately true	Selected	999,00	4,00	0,00	3,00	5,00	0,00	286,00	80,00	0,00
moderately true	Not true	moderately true	moderately true	Selected	999,00	4,00	0,00	3,00	5,00	0,00	286,00	80,00	0,00
moderately true	Not true	moderately true	moderately true	Selected	999,00	4,00	0,00	3,00	5,00	0,00	286,00	80,00	0,00
moderately true	moderately true	Not true	Not true	Selected	999,00	2,00	0,00	1,00	9,00	0,00	242,00	40,00	0,00
moderately true	moderately true	Not true	Not true	Selected	999,00	2,00	0,00	1,00	9,00	0,00	242,00	40,00	0,00
moderately true	moderately true	Not true	Not true	Selected	999,00	2,00	0,00	1,00	9,00	0,00	242,00	40,00	0,00
moderately true	moderately true	Not true	Not true	Selected	999,00	2,00	0,00	1,00	9,00	0,00	242,00	40,00	0,00
#LEEG!	#LEEG!	#LEEG!	#LEEG!	Selected	999,00	#LEEG!	#LEEG!	#LEEG!	#LEEG!	#LEEG!	#LEEG!	#LEEG!	#LEEG!
#LEEG!	#LEEG!	#LEEG!	#LEEG!	Selected	999,00	#LEEG!	#LEEG!	#LEEG!	#LEEG!	#LEEG!	#LEEG!	#LEEG!	#LEEG!
Not true	Not true	Not true	moderately true	Selected	999,00	3,00	0,00	1,00	7,00	0,00	176,00	60,00	0,00
#LEEG!	Surelly true	Not true	moderately true	Selected	999,00	3,43	1,00	1,25	7,00	0,00	176,00	68,57	0,00
Surelly true	Surelly true	Not true	moderately true	Selected	999,00	10,67	2,00	5,00	5,00	0,00	176,00	213,33	0,00
Not true	Not true	Not true	moderately true	Selected	999,00	2,29	1,00	1,00	7,00	0,00	176,00	45,71	0,00
Not true	Not true	Not true	moderately true	Selected	999,00	3,00	0,00	1,00	7,00	0,00	176,00	60,00	0,00
moderately true	Not true	Not true	Not true	Selected	999,00	1,00	0,00	0,00	9,00	0,00	770,00	20,00	0,00
moderately true	Not true	moderately true	moderately true	Selected	999,00	17,00	6,00	5,00	2,50	0,00	#LEEG!	340,00	0,00
moderately true	Surelly true	moderately true	Not true	Selected	999,00	3,00	0,00	2,00	4,00	0,00	0,00	60,00	0,00
Surelly true	Surelly true	Surelly true	Surelly true	Selected	999,00	6,00	0,00	6,00	6,00	0,00	440,00	120,00	0,00
Surelly true	Surelly true	Surelly true	Surelly true	Selected	999,00	6,40	0,00	6,00	6,00	0,00	#LEEG!	128,00	0,00
Not true	Not true	Not true	Not true	Selected	999,00	6,00	3,00	1,00	8,00	0,00	286,00	120,00	0,00
Surelly true	moderately true	Not true	Not true	Selected	999,00	21,33	10,00	2,50	8,00	1,00	110,00	426,67	0,00
moderately true	moderately true	Not true	moderately true	Selected	999,00	17,00	10,00	2,00	5,00	0,00	704,00	340,00	0,00
moderately true	Surelly true	moderately true	Not true	Selected	999,00	3,00	0,00	2,00	4,00	0,00	0,00	60,00	0,00
moderately true	moderately true	moderately true	moderately true	Selected	999,00	6,40	0,00	3,00	6,00	0,00	308,00	128,00	0,00
moderately true	moderately true	moderately true	moderately true	Selected	999,00	6,00	0,00	3,00	6,00	0,00	308,00	120,00	0,00
Not true	#LEEG!	moderately true	moderately true	Selected	999,00	4,00	0,00	3,00	6,00	0,00	264,00	80,00	0,00
Not true	moderately true	moderately true	moderately true	Selected	999,00	4,00	0,00	3,00	6,00	0,00	264,00	80,00	0,00
moderately true	moderately true	moderately true	moderately true	Selected	999,00	6,00	0,00	3,00	6,00	0,00	308,00	120,00	0,00
moderately true	moderately true	moderately true	moderately true	Selected	999,00	6,00	0,00	3,00	6,00	0,00	308,00	120,00	0,00
Not true	moderately true	moderately true	moderately true	Selected	999,00	4,00	0,00	3,00	7,00	0,00	286,00	80,00	0,00
moderately true	moderately true	moderately true	moderately true	Selected	999,00	4,00	0,00	3,00	5,00	0,00	264,00	80,00	0,00
Not true	moderately true	moderately true	moderately true	Selected	999,00	4,00	0,00	3,00	6,25	0,00	264,00	80,00	0,00

prosocialsdqR	emotionalsdqR
1,00	0,00
0,00	0,00
0,00	1,00
0,00	0,00
0,00	0,00
0,00	0,00
1,00	0,00
1,00	0,00
0,00	0,00
0,00	0,00
0,00	0,00
0,00	0,00
1,00	0,00
0,00	0,00
0,00	0,00
0,00	0,00
1,00	0,00
0,00	0,00
1,00	0,00
0,00	0,00
0,00	0,00
0,00	0,00
0,00	0,00
0,00	0,00
0,00	0,00
1,00	0,00
1,00	0,00
0,00	0,00
0,00	0,00
0,00	0,00
0,00	0,00
0,00	1,00
0,00	1,00
1,00	0,00
0,00	0,00
1,00	0,00
0,00	1,00
0,00	1,00
0,00	0,00
1,00	0,00
0,00	0,00
1,00	0,00
0,00	0,00
1,00	0,00
1,00	0,00
0,00	1,00
0,00	1,00
0,00	0,00

1,00	1,00
0,00	0,00
0,00	1,00
0,00	1,00
0,00	1,00
1,00	0,00
1,00	1,00
1,00	1,00
0,00	0,00
1,00	1,00
0,00	0,00
1,00	0,00
0,00	0,00
1,00	0,00
1,00	0,00
1,00	0,00
1,00	0,00
0,00	0,00
0,00	0,00
0,00	0,00
0,00	0,00
1,00	1,00
1,00	1,00
0,00	1,00
0,00	1,00
0,00	1,00
1,00	0,00
0,00	0,00
1,00	1,00
0,00	0,00
1,00	0,00
0,00	0,00
0,00	0,00
0,00	0,00
0,00	0,00
0,00	0,00
0,00	0,00
0,00	0,00
0,00	0,00
0,00	0,00
0,00	0,00
0,00	0,00
0,00	0,00
0,00	0,00
0,00	0,00
1,00	0,00
0,00	0,00
0,00	0,00
0,00	0,00

0,00	0,00
0,00	0,00
0,00	0,00
1,00	0,00
0,00	0,00
1,00	0,00
0,00	0,00
1,00	0,00
0,00	0,00
0,00	0,00
0,00	0,00
0,00	0,00
0,00	0,00
#LEEG!	#LEEG!
0,00	0,00
0,00	0,00
0,00	0,00
0,00	0,00
0,00	0,00
1,00	0,00
0,00	1,00
0,00	0,00
0,00	1,00
0,00	0,00
0,00	0,00
0,00	0,00
0,00	0,00
0,00	0,00
0,00	0,00
0,00	0,00
0,00	0,00
0,00	0,00
0,00	0,00
0,00	0,00
0,00	0,00
0,00	0,00
0,00	0,00
0,00	0,00
0,00	0,00
0,00	0,00
0,00	0,00
0,00	0,00
0,00	0,00
0,00	0,00
0,00	0,00
0,00	0,00
0,00	0,00
0,00	0,00
0,00	0,00

0,00	0,00
0,00	0,00
0,00	0,00
0,00	0,00
0,00	0,00
0,00	0,00
0,00	0,00
#LEEG!	#LEEG!
#LEEG!	#LEEG!
0,00	0,00
0,00	0,00
0,00	0,00
0,00	0,00
0,00	0,00
0,00	0,00
0,00	0,00
0,00	0,00
0,00	0,00
0,00	0,00
0,00	0,00
0,00	1,00
0,00	1,00
0,00	0,00
0,00	0,00
0,00	0,00
0,00	0,00
0,00	0,00
0,00	0,00
0,00	0,00
0,00	0,00
0,00	0,00
0,00	0,00
